# Type I interferon-mediated autoinflammation due to DNase II deficiency

**DOI:** 10.1038/s41467-017-01932-3

**Published:** 2017-12-19

**Authors:** Mathieu P. Rodero, Alessandra Tesser, Eva Bartok, Gillian I. Rice, Erika Della Mina, Marine Depp, Benoit Beitz, Vincent Bondet, Nicolas Cagnard, Darragh Duffy, Michael Dussiot, Marie-Louise Frémond, Marco Gattorno, Flavia Guillem, Naoki Kitabayashi, Fabrice Porcheray, Frederic Rieux-Laucat, Luis Seabra, Carolina Uggenti, Stefano Volpi, Leo A H. Zeef, Marie-Alexandra Alyanakian, Jacques Beltrand, Anna Monica Bianco, Nathalie Boddaert, Chantal Brouzes, Sophie Candon, Roberta Caorsi, Marina Charbit, Monique Fabre, Flavio Faletra, Muriel Girard, Annie Harroche, Evelyn Hartmann, Dominique Lasne, Annalisa Marcuzzi, Bénédicte Neven, Patrick Nitschke, Tiffany Pascreau, Serena Pastore, Capucine Picard, Paolo Picco, Elisa Piscianz, Michel Polak, Pierre Quartier, Marion Rabant, Gabriele Stocco, Andrea Taddio, Florence Uettwiller, Erica Valencic, Diego Vozzi, Gunther Hartmann, Winfried Barchet, Olivier Hermine, Brigitte Bader-Meunier, Alberto Tommasini, Yanick J. Crow

**Affiliations:** 1 INSERM UMR1163, Laboratory of Neurogenetics and Neuroinflammation, Paris, 75015 France; 2 Department of Medicine, Surgery, and Health Sciences, University of Trieste, Trieste, 34149 Italy; 3 Institute for Clinical Chemistry and Clinical Pharmacology, University of Bonn, Bonn, 53127 Germany; 4 Division of Evolution and Genomic Sciences, School of Biological Sciences, Faculty of Biology, Medicine and Health, University of Manchester, Manchester Academic Health Science Centre, Manchester, M13 9PT UK; 5 Bioaster, Immunomonitoring Unit, Paris, 75015 France; 6 Immunobiology of Dendritic Cells, Institut Pasteur, Paris, 75015 France; 7 INSERM U1223, Paris, 75015 France; 8 Plateforme Bio-informatique, Université Paris Descartes-Structure, Fédérative de Recherche Necker, INSERM US24/CNRS, UMS 3633, Paris, 75015 France; 9 Centre for Translational Research, Institut Pasteur, Paris, 75015 France; 10 INSERM UMR 1163, CNRS ERL 8254, Laboratory of Cellular and Molecular Mechanisms of Hematological Disorders and Therapeutical Implications, Imagine Institute, Université Paris Descartes, Sorbonne Paris-Cité et Assistance publique-Hôpitaux de Paris, Hôpital Necker, Paris, France, Laboratory of Excellence GR-ex, Paris, 75015 France; 11 Unita’ Operativa Pediatria 2, Istituto Giannina Gaslini, Genova, 16147 Italy; 12 Paris Descartes University, Sorbonne-Paris-Cité, Institut Imagine, Paris, 75015 France; 13 Laboratory of Immunogenetics of Pediatric Autoimmunity, INSERM UMR 1163, Paris, 75015 France; 14 Bioinformatics Core Facility, School of Biological Sciences, Faculty of Biology, Medicine and Health, University of Manchester, Manchester, M13 9PT UK; 15 Laboratoire d’Immunologie Biologique, Hôpital Necker-Enfants Malades, Assistance Publique-Hôpitaux de Paris, Paris, 75015 France; 16 Service d’endocrinologie, Gynécologie et Diabétologie Pédiatriques, Hôpital Necker-Enfants Malades, Assistance Publique-Hôpitaux de Paris, Paris, 75015 France; 17 INSERM U1016, Institut IMAGINE, Université Paris Descartes, Paris, 75015 France; 18 Institute for Maternal and Child Health-IRCCS “Burlo Garofolo”- Trieste, Trieste, 34137 Italy; 19 Pediatric Radiology Department, Hôpital Necker-Enfants Malades, Assistance Publique-Hôpitaux de Paris, Paris, 75015 France; 20 INSERM UMR1163, Imagine Institute, Paris Descartes University, Paris, 75015 France; 21 Department of Biological Haematology, Hôpital Necker-Enfants Malades, Assistance Publique-Hôpitaux de Paris, Paris, 75015 France; 22 Institut Necker-Enfants Malades, INSERM U1151—CNRS UMR 8253, Hôpital Necker-Enfants Malades, Assistance Publique-Hôpitaux de Paris, Paris, 75015 France; 23 Pediatric Nephrology Department, Hôpital Necker-Enfants Malades, Assistance Publique-Hôpitaux de Paris, Paris, 75015 France; 24 Pathology Department, Hôpital Necker-Enfants Malades, Assistance Publique-Hôpitaux de Paris, Paris, 75015 France; 25 Department of Advanced Diagnostic and Clinical Trials, Institute for Maternal and Child Health-IRCCS “Burlo Garofolo”, Trieste, 34137 Italy; 26 Pediatric Hepatology Unit, Hôpital Necker-Enfants Malades, Assistance Publique-Hôpitaux de Paris, Paris, 75015 France; 27 Service d’hématologie-Centre de Traitement de l’Hémophilie, Hôpital Necker-Enfants Malades, Assistance Publique-Hôpitaux de Paris, Paris, 75015 France; 28 Department of Otorhinolaryngology, Head and Neck Surgery, University Hospital Bonn, Bonn, 53105 Germany; 29 INSERM UMR_S1176, Univ. Paris-Sud, Université Paris-Saclay, Le Kremlin-Bicêtre, Paris, 94276 France; 30 Laboratoire d’Hématologie, Hôpital Necker-Enfants Malades, Assistance Publique-Hôpitaux de Paris, Paris, 75015 France; 31 Pediatric Immunology-Hematology and Rheumatology Unit, Hôpital Necker-Enfants Malades, Assistance Publique-Hôpitaux de Paris, Paris, 75015 France; 32 Study Center for Primary Immunodeficiencies, Hôpital Necker-Enfants Malades, Assistance Publique-Hôpitaux de Paris, Paris, 75015 France; 33 Necker Medical School, Paris, 75015 France; 34 INSERM UMR1163, Laboratory of Lymphocyte Activation and Susceptibility to EBV, Imagine Institute, Paris Descartes University, Paris, 75015 France; 35 Department of Life Sciences, University of Trieste, Trieste, 34128 Italy; 36 German Center for Infection Research (DZIF), Cologne-Bonn, Bonn, 53127 Germany; 37 Service d’hématologie, Faculté de Médecine Paris Descartes, Sorbonne Paris-Cité et Assistance Publique-Hôpitaux de Paris Hôpital Necker, Paris, 75015 France; 38 Department of Genetics, Hôpital Necker-Enfants Malades, Assistance Publique-Hôpitaux de Paris, Paris, 75015 France

## Abstract

Microbial nucleic acid recognition serves as the major stimulus to an antiviral response, implying a requirement to limit the misrepresentation of self nucleic acids as non-self and the induction of autoinflammation. By systematic screening using a panel of interferon-stimulated genes we identify two siblings and a singleton variably demonstrating severe neonatal anemia, membranoproliferative glomerulonephritis, liver fibrosis, deforming arthropathy and increased anti-DNA antibodies. In both families we identify biallelic mutations in *DNASE2*, associated with a loss of DNase II endonuclease activity. We record increased interferon alpha protein levels using digital ELISA, enhanced interferon signaling by RNA-Seq analysis and constitutive upregulation of phosphorylated STAT1 and STAT3 in patient lymphocytes and monocytes. A hematological disease transcriptomic signature and increased numbers of erythroblasts are recorded in patient peripheral blood, suggesting that interferon might have a particular effect on hematopoiesis. These data define a type I interferonopathy due to DNase II deficiency in humans.

## Abstract (web summary)

Nucleic acid sensing is important to ensure that an innate immune response is only mounted against microbial nucleic acid. Here, the authors identify loss-of-function mutations in the *DNASE2* gene that cause type I interferon-mediated autoinflammation due to enhanced systemic interferon signaling.

## Introduction

In 1989, Charles Janeway predicted the existence of pattern-recognition receptors (PRRs) serving to detect microbial molecular motifs distinct from self as a fundamental aspect of immunity^1^. Such motifs, which must be evolutionarily essential to the microbe so that they are not easily mutated for the ‘purpose’ of escaping a host immune response, are now recognized to include constituents of the bacterial cell wall and the nucleic acid component of viruses. In a vindication of the Janeway hypothesis, a number of PRRs have been identified, including those that detect nucleic acids in endosomal (TLR, Toll-like receptors) and cytosolic (particularly, RIG-I, retinoic-acid-inducible gene I; MDA5, melanoma differentiation-associated protein 5; AIM2, absent in melanoma 2; cGAS, cyclic GMP-AMP synthase) cellular compartments^2^. Indeed, it has become clear that most antiviral responses are initiated by innate immune receptors that detect viral nucleic acids, immediately raising the question as to how a cell discriminates between self and non-self, given that the basic molecular structure of DNA and RNA is conserved across species^3, 4^. Study of the function of these receptors has shed light on this issue, with their physical separation from self nucleic acids, the differential modification of endogenous vs. exogenous nucleic acids, thresholds of receptor tolerance for endogenous nucleic acid species and systems of self nucleic acid elimination all contributing to ensure self non-immunogenicity^5^. However, the observation of human autoimmune and autoinflammatory states indicates that these systems are not perfect, with the Mendelian type I interferonopathies^6, 7^, for example due to loss-of-function of the cytosolic DNase TREX1 signaling through the DNA sensor cGAS and its adapter molecule stimulator of interferon genes (STING)^8–12^, highlighting the potential for type I interferon induction by self-derived nucleic acid.

Using a screening assay to identify previously uncharacterized inborn errors of immunity associated with enhanced type I interferon signaling^13, 14^, we ascertain three patients from two unrelated families demonstrating a spectrum of clinical features including resolving neonatal anemia, membranoproliferative glomerulonephritis, liver fibrosis, deforming arthropathy and increased anti-DNA antibodies. We present data to show that the disease in these patients is due to biallelic loss-of-function mutations in *DNASE2*, encoding the lysosomal endonuclease DNase II, leading to an autoinflammatory state including markedly enhanced type I interferon signaling. DNase II plays a central role in the clearance of nucleic acids generated through apoptosis and the phagocytosis of maturating erythroblast nuclei, so that the absence of DNase II in mice leads to a chronic activation of type I interferon signaling mediated through the cGAS STING) pathway^15^. As such, the cases presented here further emphasize the pathological consequences of dysregulated nucleic acid sensing in the human context.

## Results

### Clinical phenotype

**Fig. 1:**
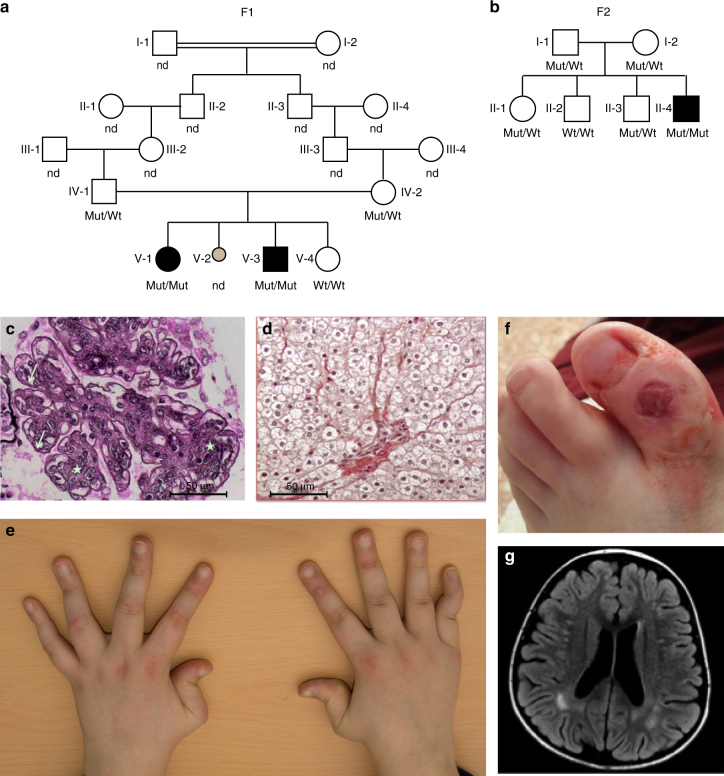
Clinical findings associated with bilallelic mutations in *DNASE2*. **a** Pedigrees of families F1 **a** and F2 **b**. Circles and squares indicate female and male family members respectively. Black symbols represent family members with a homozygous mutation in *DNASE2*. Mut mutation, Wt wild-type, ND genotype not determined. F1:V-2 denotes a fetus delivered in late pregnancy with hydrops fetalis, hepatosplenomegaly and petechiae. **c** Membranoproliferative glomerulonephritis seen on renal biopsy (×40) of F1:V-3 at age 6 years in the absence of features of lupus nephritis. There is increased lobulation and cellularity (stars) of the mesangial matrix associated with double contours (arrows). Similar findings were present in F2:II-4. **d** Liver fibrosis on biopsy (x20) of F1:V-3 at age 8 years. Similar features were also present in F2:II-4. **e** Non-destructive, deforming arthropathy seen in F2:II-4 beginning at age 8 years. **f** Vasculitic lesion on the foot of F2:II-4 at age 13 years. **g** Patchy, sub-cortical white matter lesions in the parietal lobes recorded on FLAIR imaging in F1:V-3 at age 8 years, and which were also observed in F2:II-4

By systematic screening of patients with putative inflammatory phenotypes, we ascertained a female–male sibling pair born to consanguineous parents of Algerian ancestry (Fig. 1a), and a singleton born to a European Italian couple who were not knowingly related (Fig. 1b), demonstrating increased expression of a panel of six interferon-stimulated genes (ISGs) (*IFI27*, *IFI44L*, *IFIT1*, *ISG15*, *RSAD2*, and *SIGLEC1*) (Supplementary Figs. 1 and 2). Clinical and laboratory details of the three affected individuals are provided in Table 1 and Supplementary Tables 1–5. All three children demonstrated severe non-regenerative anemia and thrombocytopenia at birth necessitating red blood cell and platelet transfusions, together with hepatosplenomegaly and cholestatic hepatitis which resolved in the first few weeks of life. All three patients also experienced recurrent fevers starting between 5 and 7 years of age, and the onset of proteinuria between the ages of 6 and 10 years, with membranoproliferative glomerulonephritis documented in the younger affected child from Family 1 (F1; F1:V-3) and the proband from Family 2 (F2; F2:II-4) (Fig. 1c). Both these children also subsequently developed hepatosplenomegaly with fibrosis identified on liver biopsy (Fig. 1d and Supplementary Fig. 3). F1:V-3 developed antibody-negative insulin-dependent diabetes mellitus at 5 years of age, as well as persistent non-regenerative anemia and hypogammaglobulinemia necessitating repeated blood transfusions and intravenous immunoglobulin beginning at 7 years of age. Starting at 8 years of age, F2:II-4 experienced the onset of a non-destructive, deforming arthropathy which has been refractory to broad-spectrum immunosuppression, anti-IL-1 receptor, anti-IL-1β and anti-TNFα therapies (Fig. 1e). At the age of 13 years, this patient also developed vasculitic skin lesions (Fig. 1f). F1:V-3 and F2:II-4 were recorded to show non-specific sub-cortical white matter lesions on cerebral imaging in the absence of overt neurological signs, although F1:V-3 exhibited learning difficulties necessitating extra help at school (Fig. 1g). At the age of 10 years, the oldest child from Family 1 (F1:V-I) is essentially clinically asymptomatic, although she has recently developed significant proteinuria. All three patients demonstrated a persistent increase in erythrocyte sedimentation rate (ESR), whilst C-reactive protein (CRP) levels were within the normal range in both affected individuals from family F1 but more frequently elevated in F2:II-4 (Supplementary Fig. 4). Serial testing also revealed fluctuating significant titers of anti-DNA antibodies in all three affected individuals.

**Table 1:** Clinical details of the three affected individuals with biallelic mutations in *DNASE2*

	F1:V-1	F1:V-3	F2:II-4
Gender (relationship)	Female (sister of F1:V-3)	Male (brother of F1:V-1)	Male
Current age	10 years	8 years	17 years
Current growth status	Height-0.2 SD; weight-0.7 SD	Height-2.2 SD; weight-0.9 SD	Height-5.95 SD; weight-4.39 SD
Features at presentation	Neonatal HSM, cholestatic hepatitis and pancytopenia requiring multiple RBC and platelet transfusions	Neonatal HSM, cholestatic hepatitis and pancytopenia requiring multiple RBC and platelet transfusions	Neonatal HSM, cholestatic hepatitis and pancytopenia requiring multiple RBC and platelet transfusions
Hematological status	Resolving neonatal pancytopenia; mild thrombocytopenia and neutropenia from age 10 years	Neonatal pancytopenia resolving by age 1 month; persistence of a mild thrombocytopenia with fluctuating neutropenia, and an episode of pancytopenia at age 4 years followed by progressive non-regenerative normocytic anemia from 7 years of age requiring recurrent blood transfusions	Neonatal pancytopenia resolving by age 2–3 months; normocytic anemia noted at age 8 years; bone marrow analysis showed normal cell composition but reduced cell numbers, considered indicative of inflammatory damage
Recurrent fevers	Starting at age 7 years, typically lasting 48 h, associated with raised ESR but essentially normal CRP	Starting at age 5 years, typically lasting 48 h, associated with raised ESR but essentially normal CRP	Starting at age 5 years, typically lasting 4–5 days, associated with swollen and painful knees, elbows, feet, wrists, raised ESR and CRP
Postnatal hepatic disease	HSM present at age 7 years with increased liver stiffness (no biopsy performed)	Longstanding HSM with fibrotic changes noted on biopsy at age 5 years	Liver biopsies performed in the neonatal period (cholestatic hepatitis), and then at the age of 3 (‘possible cirrhosis’) and 8 years (fibrosis in the absence of active inflammatory infiltrates); normal fibroscan recorded at 17 years of age
Renal status	Proteinuria recorded at age 10 years, presumed secondary to MGN but not biopsied	MGN without features of SLE at age 6 years (immunofluorescence staining negative for IgA, IgM, C3 and C1q)	Proteinuria with features of MGN diagnosed at age 8 years; normal values of C3 and C4; proteinuria no longer apparent at age 14 years, although renal biopsy showed immunocomplex deposition with abundant C1q accumulation
Neurological status	Developmentally normal	Normal early motor and cognitive milestones, now demonstrating moderate learning difficulties at school; cranial MRI at age 8 years showing patchy sub-cortical white matter hyperintensities on T2 weighted imaging, and possible subtle calcification in the basal ganglia	Headaches and mild learning difficulties; cranial MRI at age 15 years showing small sub-cortical white matter hyperintensities on T2 weighted imaging
Joint disease	None	None	Non-destructive deforming arthropathy beginning at age 8 years, particularly affecting the knees, hips, elbows and wrists, hands and temporo-mandibular joints, which has been refractory to broad-spectrum immunosuppression, anti-IL-1 receptor, anti-IL-1β and anti-TNFα therapies
Immune status	Normal; fluctuating significant elevation of anti-DNA antibodies	Transient B-cell lymphopenia at birth, recurring at age 4 years, with frank hypogammaglobulinemia from 7 years of age requiring IVIG; progressive CD4^+^ and CD8^+^ lymphopenia first recorded at age 6 years; fluctuating significant elevation of anti-DNA antibodies	Mild lymphopenia; fluctuating significant elevation of anti-DNA antibodies
Endocrinological status	Normal	IDDM from age 5 years (negative for anti-GAD, anti-IA2, anti-Langerhans islet and anti-ZnT8 antibodies)	Reduced response to arginine suggestive of growth hormone deficiency noted at age 17 years
Skin involvement	None	None	Lipodystrophy of the limbs and chilblain-like lesions of the hands and feet since the age of 13 years
Current status and treatments	Clinically asymptomatic but exhibits mild thrombocytopenia, neutropenia and proteinuria, as well as continued upregulation of ISGs and ESR; not currently treated, but due to start MMF and steroids in view of persistent proteinuria	Dependent on immunosuppression for renal disease; MMF, low dose steroids, IVIG replacement therapy, insulin, and RBC transfusions	Continued joint disease with Cushingoid features and failure to thrive, without signs of puberty; hydroxychloroquine, mepacrine, abatacept and low-dose steroids

CRP C-reactive protein; ESR erythrocyte sedimentation rate; HSM hepatosplenomegaly; IDDM insulin-dependent diabetes mellitus; ISGs interferon-stimulated genes; IVIG intravenous immunoglobulin; MGN membranoproliferative glomerulonephritis; MMF mycophenolate mofetil; MRI magnetic resonance imaging; NR not recorded; RBC red blood cell; SLE systemic lupus erythematosus

### Genetic analysis and identification of mutations in *DNASE2*

**Fig. 2:**
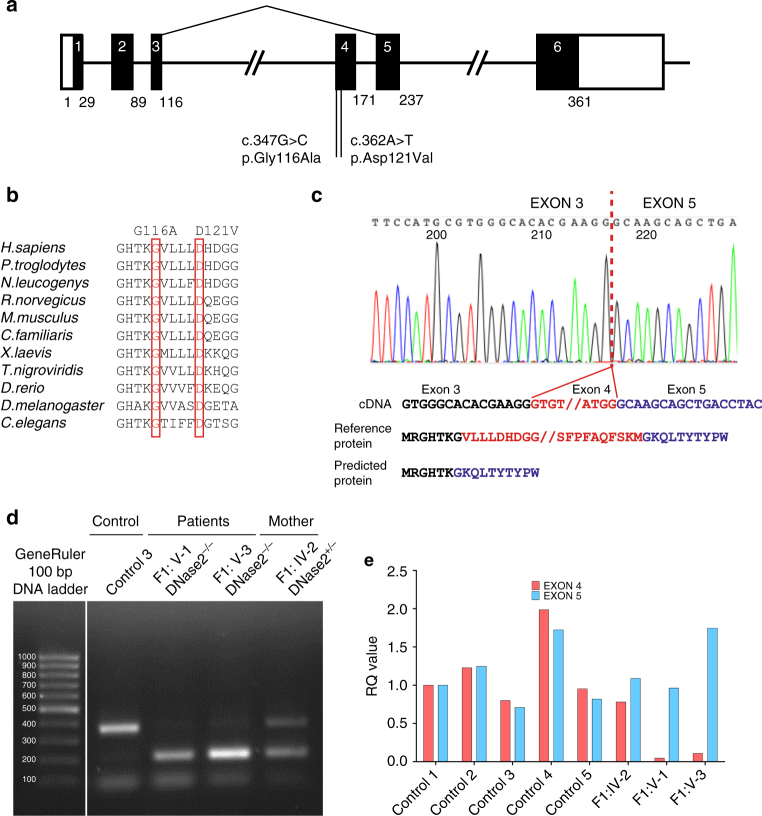
Genetic findings associated with bilallelic mutations in *DNASE2*. **a** Intron/exon structure of the *DNASE2* gene, with the splicing-out of exon 4 consequent upon the c.347G>C mutation indicated by the lines above the figure. Coding exons are represented by the black boxes, with the respective amino acid count given below each exon. The positions of the two homozygous mutations are shown. **b** A CLUSTAL Omega alignment of DNase II homologs illustrates the strict evolutionary conservation of the homozygous mutations identified in families F1 and F2 (boxed in red). **c** An electropherogram of cDNA from peripheral blood mononuclear cells (PBMCs) of F1:V-I, illustrating the loss of exon 4. **d** Gel electrophoresis of cDNA product amplified across exon 4 of *DNASE2* from a control, F1:V-1, F1:V-3 and F1:IV-2 (the mother of the two affected individuals in this family). F1:IV-2 shows a wild-type band as observed in the control, and a smaller band as seen in her homozygous mutant offspring. A second, fainter, wild-type band is also present in the affected patients, best seen after longer exposure, likely representing a degree of leaky splicing. **e** qPCR of cDNA using a TAQman probe specific for exon 4 compared to a probe for exon 5 of *DNASE2* supports this assertion, where a small amount of exon 4 message was detected in peripheral blood mononuclear cells (PBMCs) from F1:V-1 and F1:V-3

We performed whole-exome sequencing using DNA from the three patients and their parents, and we filtered coding variants against allele frequencies in public and local databases. We recorded a homozygous c.347G>C variation in *DNASE2* in the two affected individuals from family F1, and a homozygous c.362A>T variant in the same gene in the proband of family F2 (Fig. 2a). Both sets of parents were heterozygous for the relevant familial variant, and all four unaffected siblings from both families were either homozygous or heterozygous wild type (Supplementary Fig. 5).

The c.347G>C transversion leads to the substitution of a glycine for an alanine at amino acid position 116 (p.Gly116Ala/G116A) of the human DNase II protein, whilst the c.362A>T transversion results in the substitution of an aspartate by a valine at position 121 (p.Asp121Val/D121V). The glycine residue at 116 and the aspartate residue at 121 are highly conserved (Fig. 2b and Supplementary Fig. 6), and both substitutions, neither of which is recorded on the gnomAD database comprising >245,000 alleles at these positions, are predicted as damaging according to a variety of in silico algorithms (Supplementary Table 6 and Supplementary Fig. 7).

The G at base 347 of the cDNA is the first nucleotide of exon 4, and is thus predicted to act as an acceptor for RNA splicing, a process likely affected by the G>C transversion^16^. Sequencing of cDNA from peripheral blood mononuclear cells (PBMCs) of F1:V-1 and F1:V-3 confirmed an in-frame deletion of exon 4, encoding amino acids 116 to 171 (Fig. 2c). In the two affected siblings, gel electrophoresis of cDNA product amplified across exon 4 of *DNASE2* revealed a shorter band compared to controls, as well as a faint wild-type band. F1:IV-2 demonstrated a wild-type band and a band at the same size as seen in her two affected children, consistent with her heterozygous status (Fig. 2d). qPCR of cDNA using TAQman probes specific for exons 4 and 5 of *DNASE2* supported these data, suggesting a degree of leaky splicing and the production of some non-deleted product in F1:V-1 and F1:V-3 (Fig. 2e). Furthermore, sequencing of the faint band of wild-type size obtained by PCR of cDNA from F1:V-1 indicated the presence of full-length transcript including the c.347G>C variant (Supplementary Fig. 8). Western blot analysis of macrophage-enriched cells from F1:IV-2 revealed only a single band at the size of the full-length protein, indicating that the deleted product was not translated or was unstable in this cell type (Supplementary Fig. 9). Sufficient material was not available for a similar analysis in the affected patients, and blotting of protein extracts derived from fibroblasts did not produce interpretable data.

### Mutations in *DNASE2* result in a loss of DNase II activity

**Fig. 3:**
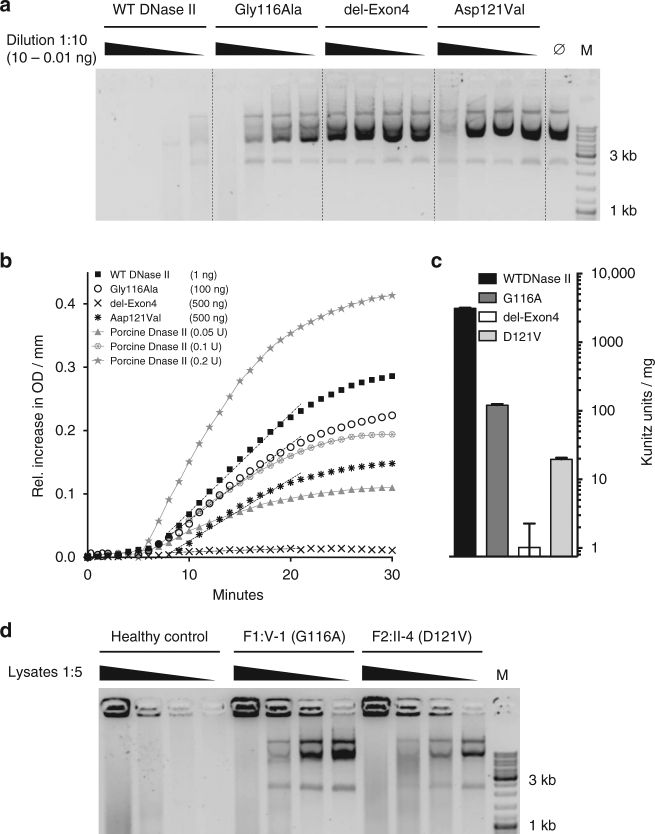
Effect of *DNASE2* mutations on DNase II activity. **a** DNase II activity of constructs expressed in HEK293T cells against circularized plasmid DNA. Titration of purified protein incubated with plasmid DNA for 1 h. **b** Quantification of DNase II activity via absorption at 260 nm. The gray lines denote assay calibration with porcine DNase II. **c** DNase II activity is calculated via linear approximation according to the Kunitz protocol (WT 3108 Ku/mg; G116A 120 Ku/mg; del-Exon4 0 Ku/mg; D121V 19.57 Ku/mg). Linear approximation was performed with PRISM6. Data are shown as the mean ± SD (*n* = 3). These data are representative of two independent experiments. **d** DNase II activity against circularized plasmid DNA recorded in fibroblasts from F1:V-1 and F2:II-4 compared to cells from a healthy control, which could be rescued by transfection of a construct coding for wild-type (WT) protein

The aspartate at position 121 falls within the N terminal phospholipase D domain which, together with the histidine at position 130, likely plays an important role in DNase II catalytic function^17^. Both of these residues are encoded by exon 4 of *DNASE2*, and would therefore be absent in the mis-spliced transcripts resulting from the c.347G>C mutation in family F1 (Supplementary Fig. 10). Expression in HEK293T cells of mutant constructs lacking exon 4, or with the c.347G>C (Gly116Ala) or the c.362A>T (Asp121Val) substitutions, was associated with reduced levels of DNase II activity against circularized plasmid DNA compared to controls (WT) (Fig. 3a–c). Furthermore, lysates of fibroblasts from F1:V-1 and F2:II-4 demonstrated a marked reduction of DNase II activity against the same substrate; DNase II activity in these cells could be restored by expression of constructs coding for wild-type protein (Fig. 3d). Concordant data were obtained after siRNA knockdown of DNASE2 in control fibroblasts (Supplementary Fig. 11). Taken together, these data provide validation of the pathogenicity of the *DNASE2* variants recorded in each family.

### Loss of DNase II activity induces interferon signaling

DNase II null mice accumulate undigested DNA in the lysosomes of macrophages which then chronically activates type I interferon production to result in a lethal perinatal anemia^18–20^. In contrast, *DNase2* knockout mice also lacking the type I interferon receptor (Ifnar1) live beyond birth, but develop a cytokine-dependent chronic polyarthritis^21, 22^. Both the embryonic and post-natal phenotypes are cGAS and STING-dependent^12, 23^.

**Fig. 4:**
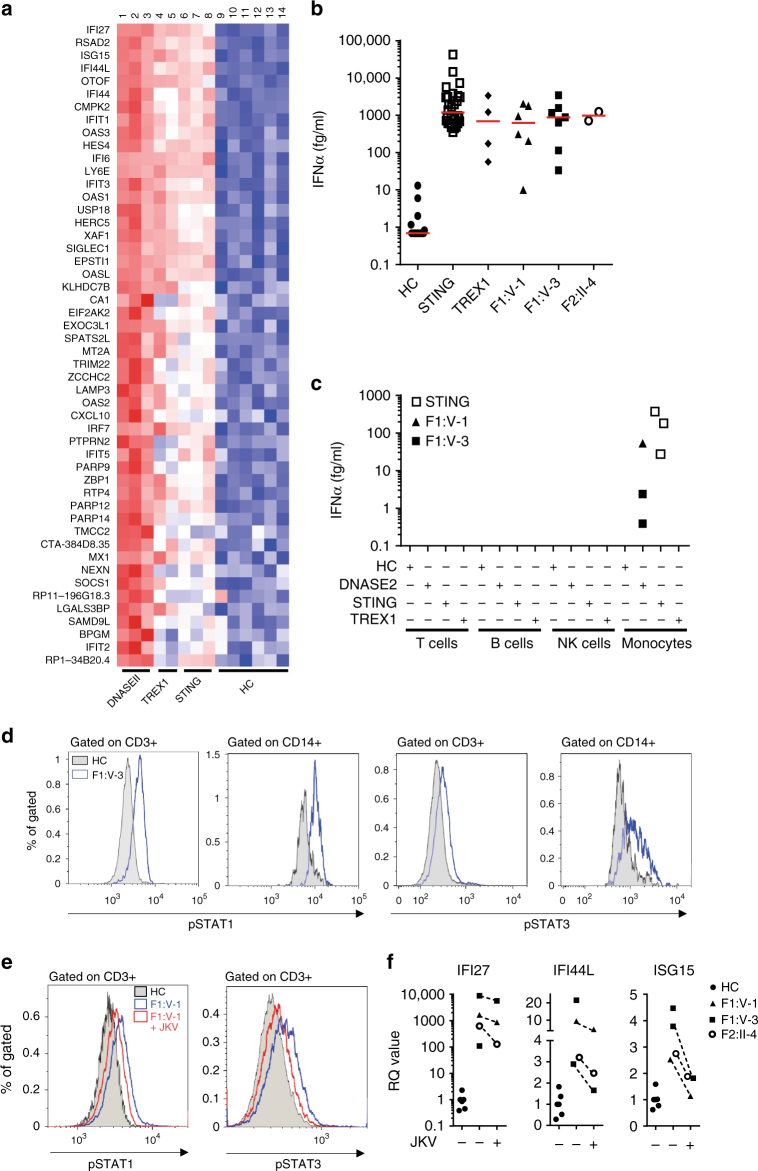
Interferon signaling in patients with mutations in *DNASE2*, *STING* and *TREX1*, and in controls. **a** Heat map derived from RNA-Seq expression data of the top 50 genes ranked by *p* value (*DNASE2*-mutated patients vs. controls) with the most significant at the top. 41 of these 50 genes are considered as interferon-stimulated. Lanes 1-14 show individual samples: Lane 1 *DNASE2* F1:V-1; lane 2 *DNASE2* F1:V-3; lane 3 *DNASE2* F1:V-3; lane 4 *TREX1* P1; lane 5 *TREX1* P2; lane 6 *STING* P1; lane 7 *STING* P2, lane 8 *STING* P3; lanes 9–14 individual healthy controls (HC). **b** Levels of interferon alpha (IFNα) protein assayed by digital ELISA in plasma or serum from healthy controls (HC: *n* = 20), patients with mutations in *STING* (*n* = 28 samples from 8 patients), patients with mutations in *TREX1* (*n* = 4 samples from 4 patients), F1:V-1 (6 samples taken over 3 years), F1:V-3 (7 samples taken over 3 years) and F2:II-4 (2 samples taken over 3 years). Red lines indicate median values. **c** Analysis of IFNα production by cultured T cells, B cells, natural killer (NK) cells or monocytes from controls (HC; *n* = 4), and patients with mutations in *DNASE2* (F1:V-1 1 sample; F1:V-3 2 samples), *STING* (*n* = 3) or *TREX1* (*n* = 1). **d** Increased phosphorylation of STAT1 and STAT3 observed in unstimulated CD3 positive T cells and CD14 positive monocytes from total blood of F1:V-3 compared to a healthy control (HC). **e** Increased phosphorylation of STAT1 and STAT3 observed in cultured lymphocytic-enriched fractions from F1:V-1 compared to a healthy control (HC), treated or not with the JAK1/2 inhibitor ruxolitinib. Similar results were obtained with cells from F1:V-3. **f** Increased expression of selected interferon-stimulated genes in cell fractions enriched for lymphocytes from F1:V-1, F1:V-3 and F2:II-4 with or without ruxolitinib

Whole-genome expression analysis of RNA (RNA-Seq) confirmed the results of our screening assay, demonstrating a global upregulation of ISGs in whole blood from F1:V-1 and F1:V-3 as seen in patients with gain-of-function mutations in STING or loss of TREX1 (DNase III) activity (Fig. 4a and Supplementary Table 7). Combining high-affinity pan-interferon alpha antibodies with single-molecule array (Simoa) digital ELISA technology^24, 25^, we recorded increased levels of interferon alpha protein in serum and plasma from all three patients, comparable to levels observed in other type I interferonopathies^26^ (Fig. 4b). Overexpression of the ISGs *IFIT1*, *IFI16* and *IFI27* in unstimulated patient fibroblasts could be reversed by lentiviral transduction of wild-type DNase II (Supplementary Fig. 12), and siRNA knockdown of *DNASE2* in control fibroblasts resulted in increased ISG expression similar to that observed in patient cells (Supplementary Fig. 13). As has been described for patients mutated in *TMEM173* ^26^, an analysis of cultured cell subpopulations indicated that monocytes were a major source of circulating interferon (Fig. 4c). Unstimulated CD3 positive T cells and CD14 positive monocytes demonstrated increased phosphorylation of STAT1 and STAT3 (Fig. 4d and Supplementary Fig. 14). Treatment of a cell fraction enriched for patient lymphocytes (non-adherent cells) led to a reduction of STAT1 and STAT3 phosphorylation (Fig. 4e), and a decrease in ISG expression in both adherent and non-adherent (monocyte and lymphocyte enriched, respectively) cells (Fig. 4f and Supplementary Figs. 14 and 15).

### Loss of DNase II activity results in an inflammatory state

**Fig. 5:**
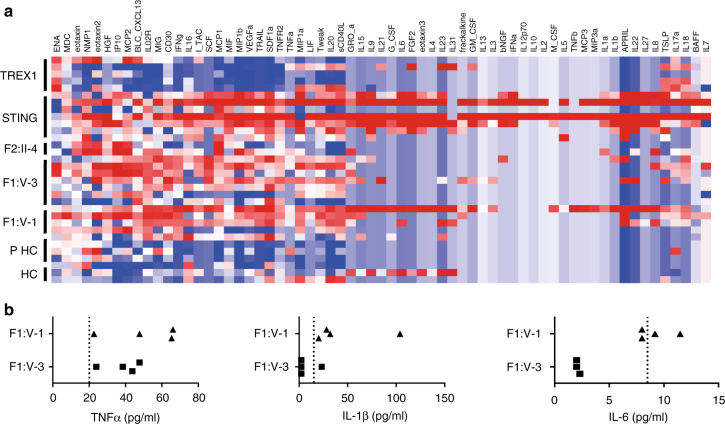
Non-interferon signaling in patients with mutations in *DNASE2*, *STING* and *TREX1*, and in controls. **a** Multiplex cytokine analysis of serum from five patients (five samples) with loss-of-function mutations in *TREX1*, 7 patients (seven samples) with gain-of-function mutations in *STING*, FII:II-4 (two samples), F1:V-3 (seven samples), F1:V-1 (five samples) and six healthy controls (comprising three children, PHC, and three adults, HC). A pattern of enhanced expression of cytokine/chemokine proteins shared across patients mutated in *STING* and *DNASE2* is indicated by the dotted lines, with genes ranked according to Spearman’s correlation. **b** Levels of TNFα, IL-1β and IL-6 protein in plasma from F1:V-1 and F1:V-3 sampled across 25 months. The dotted lines represent the cutoff for the normal range in controls for each cytokine

Mouse data emphasize the induction of both interferon and non-interferon-mediated inflammation in the absence of DNase II, highlighting a role for TNFα, IL-1β, and IL-6 in the pathology of the postnatal arthritis observed in the *DNase2* ^−/−^ *Ifnar1* ^−/−^ model^21, 22^. Multiplex analysis using serum from F1:V-1, F1:V-3 and F2:II-4 demonstrated an overlap of cytokine and chemokine protein expression induced by loss of DNase II activity and activation of STING, distinct from the inflammatory state related to mutations in TREX1 (Fig. 5a). Single analyte ELISA assays using serum from F1:V-1 and F1:V-3 were consistent with the multiplex analysis, suggesting consistently elevated protein levels of TNFα in both patients, a more variable elevation of IL-1β, and concentrations of IL-6 mostly reflecting normal physiological levels (Fig. 5b).

### Effects of DNase II loss of function on erythropoiesis

**Fig. 6:**
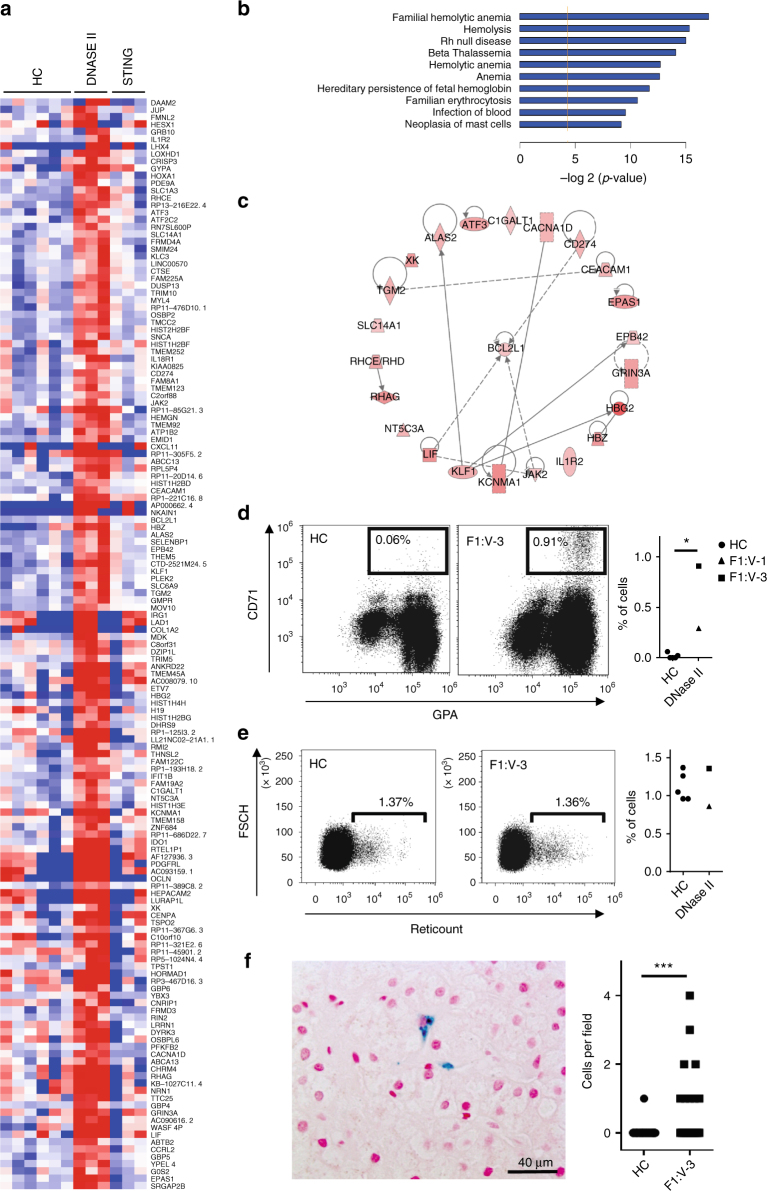
Effects of *DNASE2* mutations on red blood cell homeostasis. Heat map of genes derived by statistical analysis of RNA-Seq data selected according to the following criteria: Control vs. DNase II fold change >2 or <−2 (Adj *p* < 0.05) and STING vs. Controls (Adj *p* > 0.05) and DNase II vs. STING; (Adj *p* < 0.05). **b** Histogram of the 10 hematological functions identified as the most significantly enriched by Ingenuity Pathway Analysis (IPA) of the genes derived in **a**. Genes included in these networks are annotated in **c** with known interactions symbolized by the dotted lines. **d** Representative dot plot of a CD71 GPA staining of blood from F1:V-3 vs. a healthy control (HC). Patients (*n* = 2) demonstrated an increased proportion of circulating CD71^+^, GPA^+^ erythroblasts compare to HC (*n* = 5). **e** Representative dot plot of a reticulocyte staining of blood from F1:V-3 vs. a healthy control. Reticulocyte percentage is comparable between patients (*n* = 2) and controls (*n* = 5). **f** Liver biopsy (x40) taken from F1:V-3 at age 8 years demonstrating increased numbers of enlarged Kupffer cells containing hemosiderin (identified by Perls’ stain). Quantification of the number of such cells per field in F1:V-3 (*n* = 20) compared to 2 age-matched non-inflammatory controls (HC) (*n* = 41) is given on the right (Mann–Whitney test, *p < 0.05; ***p < 0.001)

Mammalian definitive erythropoiesis takes place in the fetal liver and bone marrow, where macrophages digest nuclei extruded by developing erythroblasts^27, 28^. *DNase2* knockout mice die in late embryogenesis or immediately after birth due to a severe anemia. Analysis of fetal liver in these mice reveals the accumulation of undigested DNA from erythroblasts, and in tissues where apoptosis occurs during development increased numbers of macrophages filled with DNA are detected^18, 19^. In keeping with this, all three children reported here demonstrated severe anemia and thrombocytopenia at birth necessitating red blood cell and platelet transfusions. Network pathway analysis of RNA-Seq data obtained from F1:V-1 and F1:V-3, excluding over-expressed ISG transcripts common to other type I interferonopathies, highlighted changes in the expression of genes related to erythropoiesis compared to controls and patients with gain-of-function mutations in STING (Fig. 6a–c and Supplementary Fig. 16). Fresh blood samples from F1:V-1 and F1:V-3 demonstrated a marked increase in the percentage of circulating erythroblasts, whilst the number of reticulocytes was the same as in controls (Fig. 6d, e and Supplementary Fig. 17). These features were suggestive of ineffective erythropoiesis, as confirmed by bone marrow cytology in F1:V-3 (Supplementary Table 8). Of note, examination of a liver biopsy from F1:V-3 revealed increased numbers of Kupffer cells staining for hemosiderin, indicating increased phagocytosis of red blood cells (Fig. 6f).

## Discussion

We describe here a multisystem autoinflammatory syndrome due to biallelic hypomorphic mutations in *DNASE2*, the gene encoding the 360 amino acid lysosomal DNase DNase II^29^. Each mammalian cell carries around 6 pg of nuclear DNA, and it has been estimated that in humans more than 1 g of cellular DNA is eliminated and degraded each day^30^. A series of elegant studies in mice has shown that DNase II digests the DNA of apoptotic cells and of nuclei expelled from erythroid precursors. DNase II null mice accumulate DNA in the lysosomes of fetal liver Kupffer cells and bone marrow macrophages^20, 27^, which activates type I interferon production resulting in a lethal anemia^18–20^. *Dnase2* knockout mice also lacking the type I interferon receptor^21^, or IRF3 and IRF7^31^, live beyond birth, but develop a chronic polyarthritis. Importantly, these embryonic and post-natal phenotypes are each rescued by crossing the DNase II null mouse against mice defective in STING^23^ or cGAS^12^, thus highlighting the central role of TLR-independent cytosolic DNA signaling in either situation.

All three children that we ascertained presented in the neonatal period with life-threatening anemia and thrombocytopenia, which resolved spontaneously within a few weeks of birth. A similar pattern of hematological involvement has been noted in patients with pseudo-infection syndromes due to loss-of-function mutations in *TREX1* ^32^ and *USP18* ^33^. Furthermore, studies in mice have implicated endogenously produced type I interferon in the depletion of hematopoietic progenitors during virus-induced transient pancytopenia^34^. These observations, together with the experimental data presented here, including the finding that recombinant interferon alpha treatment of erythroid progenitors derived from cord blood results in increased apoptosis (Supplementary Fig. 18), suggest a particular sensitivity of both murine and human fetal erythropoiesis to the abnormal production of type I interferon by DNase II-deficient macrophages. F1:V-3 also developed a later-onset anemia necessitating repeated blood transfusions. Of possible relevance, in both F1:V-1 and F1:V-3 we observed increased numbers of circulating erythroblasts suggestive of ineffective erythropoiesis, whilst circulating reticulocyte counts were inappropriate for the level of circulating hemoglobin. The mechanism by which loss of DNase II function disturbs adult erythropoiesis will require further exploration.

The phenotype associated with biallelic mutations in *DNASE2* is remarkable for its breadth. After the neonatal period, all three patients experienced recurrent febrile episodes, and both F1:V-3 and F2:II-4 demonstrated fibrosing hepatitis and membranoproliferative glomerulonephritis. Features seen in a single patient include the deforming arthropathy and vasculitic skin lesions affecting F2:II-4, while F1:V-3 developed autoantibody-negative diabetes mellitus, hypogammaglobulinemia and later-onset anemia necessitating long-term insulin replacement, intravenous immunoglobulin and blood transfusions respectively. In marked contrast to her younger brother, F1:V-I is essentially clinically asymptomatic at the age of 10 years, although she has recently developed significant proteinuria and demonstrates a chronic elevation of ESR and interferon-induced gene transcripts.

Using a new digital ELISA assay, we recorded increased levels of interferon alpha protein in serum and plasma from all three patients with mutations in DNase II, and showed that cells from these patients produce increased amounts of type I interferon and demonstrate enhanced STAT1 phosphorylation. Concordantly, we recorded a significant type I interferon signature by genome-wide RNA-Seq analysis, similar to that observed in patients with other type I interferonopathies. Although mouse data emphasize the induction of both interferon and non-interferon-mediated inflammation in the absence of DNase II, the post-natal murine phenotype associated with DNase II deficiency in the context of functional type I interferon signaling has not been defined. By multiplex analysis we identified increased expression of a number of cytokines in patient blood, with a similar pattern of expression observed in patients with gain-of-function mutations in STING. Thus, the relative contribution of interferon and non-interferon dependent pathways to the disease reported here remains unclear. F2:II-4 has experienced a severe deforming arthropathy possibly reminiscent of the *Dnase2* ^−/−^ *Ifnar1* ^−/−^ mouse model^21^. However, whilst breeding of *Dnase2* ^−/−^ *Ifnar1* ^−/−^ mice on a TNFα or IL-6 null background, or treatment with a monoclonal antibody against the IL-1 receptor, ameliorated the murine polyarthritis phenotype^22^, the use of biologics targeting TNFα and IL-1 signaling has shown minimal clinical efficacy in this patient. Of note, significant joint involvement highly reminiscent of that observed in F2:II-4 has been seen in patients with gain-of-function mutations in MDA5^35^ (Supplementary Fig. 19).

Beyond infection, systemic lupus erythematous (SLE) was the first human disease to be associated with increased type I interferon signaling^36^, and mutations in three distinct DNases, DNase I^37^, DNase III (TREX1)^38^ and DNase1L3^39, 40^, have been reported to predispose to the development of lupus. None of the patients described here fulfill criteria for a diagnosis of SLE. However, immunocomplex deposition with C1q accumulation was noted in a renal biopsy of F2:II-4, and all three patients demonstrated fluctuating elevations of anti-DNA antibodies. Given the current young age of these patients, we cannot rule out the possibility that they will develop a more complete lupus phenotype in the future.

The role of foreign nucleic acid in the induction of type I interferon was recognized over 50 years ago^41^. More recently, the importance of mechanisms to avoid the mis-sensing of self nucleic acid as non-self has been highlighted by the characterization of the Mendelian type I interferonopathies^3, 7^. While perinatal hematological disturbance, skin vasculitis and non-deforming arthropathy have been observed in certain type I interferonopathies, the disorder that we describe here represents a distinct clinical syndrome. No test to assess type I interferon status is currently available in routine medical practice, so that the phenotypic spectrum associated with enhanced type I interferon signaling will likely broaden as systematic screening, like that employed here, enters the clinical sphere^13, 26^. Phenotypic differences do not rule out the possibility of a central role for disturbed type I interferon signaling in these various disorders, but might be explained by differential tissue and temporal expression. On the other hand, the *Dnase2* ^−/−^ mouse clearly indicates both interferon and non-interferon dependent pathology, so that it is possible that clinical differences between the disorders currently classified as type I interferonopathies might also reflect differential involvement of distinct inflammatory pathways. Clarification of these issues, including how closely individual mouse models recapitulate their respective human genotypes, will likely become of increasing relevance as therapies directly targeting type I interferon signaling are developed^42–44^.

## Methods

### Patient and study approval

The study was approved by the Comité de Protection des Personnes (ID-RCB/EUDRACT: 2014-A01017-40) in France, and the Comitato Indipendente di Bioetica, IRCCS Burlo Garofolo (19/2015; RC17/2014) in Italy. Investigations were undertaken with written informed parental consent, and the assent of the probands in both families.

### Whole-exome sequencing

DNA was extracted from whole blood samples using an in-house salting out method. Agilent SureSelect libraries were prepared from 3 µg of genomic DNA extracted from leukocytes, sheared with a Covaris S2 Ultrasonicator as recommended by the manufacturer. We used a 51 Mb SureSelect Human All Exon kit V5 (Agilent technologies) for exome capture, with multiplexed molecular barcodes for sample traceability. Pooled barcoded exome libraries were then sequenced on a HiSeq2500 (Illumina) machine, generating 76 + 76 bp paired-end reads, followed by processing using Genome Analysis Toolkit (GATK), SAMtools, and Picard (http://www.broadinstitute.org/gatk/guide/topic?name = best-practices). Calls were made with the GATK HaplotypeCaller, and variants assessed using the in silico programs SIFT (http://sift.jcvi.org) and Polyphen2 (http://genetics.bwh.harvard.edu/pph2/), and population allele frequencies obtained from the ExAC (http://exac.broadinstitute.org) and gnomAD (http://gnomad.broadinstitute.org) databases.

We have screened close to 1000 probands for an interferon signature, and performed over 2500 interferon signatures in total (as outlined in Rice et al.^14^). Not every patient has had genetic analysis, and it is possible that we are not comprehensively ascertaining *DNASE2*-mutated patients because, for example, they are dying due to the perinatal hematological phenotype. However, the clinical scenario that we describe in this manuscript is apparently rare in our cohort.

### Sanger sequencing

Sanger sequencing was performed on DNA from all three patients and their parents to confirm the *DNASE2* variants found by exome sequencing. The reference sequence used for primer design and nucleotide numbering was *DNASE2* (NM_001375.2). The exonic regions and flanking intronic sites of the gene were amplified by polymerase chain reaction (PCR) using specific primers (eurofinsgenomics) designed with Primer3plus (http://www.bioinformatics.nl/cgi-bin/primer3plus/primer3plus.cgi) (Family 1 primers: *DNASE2*_e3For 5′-TGGCCAGGCTGATTTCTAAC-3′ and *DNASE2*_e4Rev 5′-GACAAGGGGAGGAGGAAATG-3′; Family 2 primers: *DNASE2*_4For 5′-GTAGAGATGGGGTTTCGCC-3′ and *DNASE2*_5Rev 5′-CAAGGGGAGGAGGAAATGC-3′).

### Liver histology

Fragments of core needle liver biopsy from F1:V-3 were fixed in buffered formalin, embedded in paraffin, cut into 3 µm thick sections and stained with hematoxylin, eosin, saffron, Perls’ (equal parts mixture of ferrocyanide and hydrochloric acid than counterstained with filtered neutral red stain), Masson’s trichrome (stepwise staining in Weigert’s Iron Hematoxylin solution, Biebrich Scarlet-Acid Fuchsin solution, phosphomolybdic-phosphotungstic acid solution and aniline blue solution), Sirius red (0.1% solution of Sirius red in saturated picric acid and then washed in 0.5% acetic acid) and reticulin (0.5% potassium permanganate followed by 2% potassium metabisulfite, 2% ferric ammonium sulfate, ammonia silver nitrate solution, 20% unbuffered formalin, 0.2% gold chloride, 2% potassium metabisulfite and 2% sodium thiosulfate). A comparison of the number and size of Kupffer cells was made between the patient and three age-matched controls whose histology was normal.

### Kidney histology

Biopsy material from F1:V-3 was fixed in buffered formalin, dehydrated in graded alcohols, embedded in paraffin, cut into 3 µm thick and treated with hematoxylin and eosin, Jones methenamine silver, Masson trichrome, or periodic acid-Schiff reagent.

### Splicing defect characterization

Total RNA was extracted from peripheral blood mononuclear cells (PBMCs) using RNAqueous-Micro Kit (Ambion). Reverse transcription was performed using the High-Capacity cDNA Reverse Transcription Kit (Applied Biosystems). For visualization of mutation-induced defective splicing, cDNA from patient and control PBMCs was amplified with primers encompassing exon 4 of human *DNASE2* (*DNASE2*_e3For 5′-TGCCAGCTCTTAGAGGGT-3′ and *DNASE2*_e5Rev 5′-AGGGTTCTTGGCTAACGTGGTG-3′). The faint band of wild-type size was purified from the gel using MicroElute Gel Extraction Kit from Omega, cloned in a pGEMT vector from Promega and sequenced by Sanger sequencing using the same primer pair as above. For exon 4 and exon 5 in family F1, quantitative reverse transcription polymerase chain reaction (qPCR) analysis was performed using TaqMan Universal PCR Master Mix (Applied Biosystems) on a ViiA 7 Real-Time PCR system, and cDNA derived from 40 ng total RNA. Using TaqMan probes for *DNASE2* exon 4 (Hs00172391_m1) and *DNASE2* exon5 (Hs00923081_m1), the relative abundance of each target transcript was normalized to the expression level of *HPRT1* (Hs03929096_g1) and 18 S* rRNA* (Hs999999001_s1), and assessed with the Applied Biosystems StepOne Software v2.1 and DataAssist Software v.3.01.

### ISG RNA expression in total blood

Whole blood was collected into PAXgene tubes. We used a PreAnalytix RNA isolation kit and RNA concentration, assessed with a spectrophotometer (FLUOstar Omega, Labtech), to extract total RNA. We performed quantitative reverse transcription polymerase chain reaction (qPCR) analysis using the TaqMan Universal PCR Master Mix (Applied Biosystems), and cDNA derived from 40 ng total RNA. Using TaqMan probes for *IFI27* (Hs01086370_m1), *IFI44L* (Hs00199115_m1), *IFIT1* (Hs00356631_g1), *ISG15* (Hs00192713_m1), *RSAD2* (Hs01057264_m1), and *SIGLEC1* (Hs00988063_m1), the relative abundance of each of 6 ‘interferon score’ target transcripts was normalized to the expression level of *HPRT1* (Hs03929096_g1), and *18* *S rRNA* (Hs999999001_s1), assessed using the Applied Biosystems StepOne Software v2.1 and DataAssist Software v.3.01. Individual data were expressed relative to a single calibrator for each of the six probes. RQ (relative quantification) is equal to 2^−^^ΔΔCt^, i.e., the normalized fold change relative to the control data. The median fold change of the 6 genes compared to the median of 29 previously collected healthy controls is used to create an interferon score for each individual, with an abnormal interferon score being defined as greater than +2 SD above the mean of the control group, i.e., 2.466.

For the extended interferon analysis, the relative abundance of target transcripts, was measured using the additional TaqMan probes *Ly6E* (Hs00158942_m1), *MX1* (Hs00895598_m1), *USP18* (Hs00276441_m1), *OAS1* (Hs00973637_m1), *IFI44* (Hs00197427_m1), *IFI6* (Hs00242571_m1), *IFIT3* (Hs00155468_m1), *IRF7* (Hs00185375_m1) and *STAT1* (Hs01013989_m1), was normalized to the expression level of *HPRT1* (Hs03929096_g1) and *18 s* (Hs999999001_s1) and assessed with Applied Biosystems StepOne Software v2.1.

### ISG RNA expression in PBMCs

Messenger RNA from treated and non-treated cultured cells were quantified by qRT-PCR using TaqMan probes for *IFI27* (Hs01086370_m1), *IFI44L* (Hs00199115_m1), *ISG15* (Hs00192713_m1), and *SIGLEC1* (Hs00988063_m1), the relative abundance of each target transcript normalized to the expression level of *HPRT1* (Hs03929096_g1), and assessed with the Applied Biosystems StepOne Software v2.1.

### ISG RNA expression analysis

1 × 10^6^ cells were lysed in 350 µL RLT-Buffer (Qiagen), frozen at −80 °C, thawed, supplemented with 1 volume 70% Ethanol and loaded onto a Zymo III column (Zymo). The column was then washed with RW1buffer (Qiagen) and then RNA wash buffer (Zymo) and eluted with DEPC water. Samples were then subjected to DNase I digestion and reverse transcription with OligodT primers and RevertAid (all from ThermoFischer, Waltham, MA, USA) according to the manufacturer’s instructions. RT-PCR was performed using with 5x EvaGreen QPCR-Mix II (ROX) (Biobudget, Krefeld, Germany). Primers for IFIT1, IFI16 and IFI44 were designed using NCBI primer blast. Sequences used were: *IFIT1* forward 5′-GTGCTTGAAGTGGACCCTGA-3′, *IFIT1* reverse 5′-CCTGCCTTAGGGGAAGCAAA-3′, *IFI16* forward 5′-ATATCCTTCAGAGGCCAGCA-3′, *IFI16* reverse 5′-ATCTGAGGAGTGTGGGGATG-3′, *IFI27* forward 5′- TGCCCATGGTGCTCAGTG-3′ and *IFI27* reverse 5′- GAGAGTCCAGTTGCTCCCAG-3′. Primers were validated with melting curve and agarose gel analysis and tested for efficiency using cDNA dilution.

### Gene expression analysis by RNA sequencing

Whole transcriptome expression analysis was performed using one sample from F1:V-1 and two samples from F1:V-3 plus three age-matched controls, three patients mutated in *TMEM173* and two patients mutated in *TREX1* (Supplementary Table 5). We extracted total RNA from blood samples in PaxGene tubes (Qiagen, Valencia, CA), and analyzed RNA integrity with an Agilent 2100 Bioanalyzer. We used the Illumina TruSeq RNA Sample Preparation Kit (Illumina) for mRNA purification and fragmentation, complementary DNA synthesis and target amplification. cDNA libraries were then pooled and sequenced on a HiSeq 2000 Illumina platform (Illumina). The RNA-seq workflow was as follows: quality assessment by FastQC (http://www.bioinformatics.babraham.ac.uk/projects/fastqc/), quality filtering with Trimmomatic (PMID: 24695404), read mapping with STAR (PMID: 23104886) to hg38, read counting into genes with HTSeq (PMID: 25260700) using annotation from GENCODE v24 (http://www.gencodegenes.org/), normalization and differential expression analysis with DESeq2 (PMID: 25516281). Hierarchical clustering was conducted using the Partek software (version 6.6). Patients and healthy controls were compared using ANOVA analysis, and a list of the 2-fold up or downregulated genes was generated. The statistical significance level adopted was *p* < 0.05. Gene lists were uploaded into Ingenuity Pathway Analysis (http://www.ingenuity.com) in order to determine differentially regulated canonical pathways in the patients. Results for interferon-regulated genes are shown as a heat map, with shades of red denoting upregulated genes and shades of blue denoting downregulated genes.

### siRNA knockdown of *DNASE2*

siRNA knockdown was performed in primary fibroblasts via repetitive transient transfection using with 150 nM of SMARTpool ON-TARGETplus siRNA (Dharmacon) targeting *DNASE2* (NCBI refseq: NM_001375) or a negative control. siRNAs were transfected via complexation with Lipofectamine 2000 (Life Technologies) according to the manufacturer’s instructions. Fibroblasts were transfected twice, with the second transfection after 48 h. DNase 2 assays were performed 24 h after the second transfection. Knockdown efficiency was evaluated by quantitative real-time PCR. The *DNASE2* primers used were: (forward) 5′-TCGCCTTCCTGCTCTACAAT-3′ and (reverse) 5′-CCCATCTTCGAGAACTGAGC-3′.

### Plasmids

Human DNase II gene ORF cDNA clone expression plasmid with a C-terminal Myc-FLAG tag, pCMV6-RC209573, was purchased from OriGene (OriGene Technologies, Rockville, Maryland, USA). The deletion of exon 4 and the mutations c.347G>C and c.362A>T were introduced into pCMV6-RC209573 via site-directed mutagenesis. The deletion of exon 4 was performed using the Q5 Site-Directed Mutagenesis Kit (New England Biolabs, Ipswich, MA, USA) according to the manufacturer’s instructions. The point mutations were introduced via whole plasmid PCR with Pfu Ultra (Stratagene). The primers used were: (deletion exon 4 sense) 5′-GCAAGCAGCTGACCTACAC-3′, (deletion exon 4 antisense) 5′-CCTTCGTGTGCCCACGCA-3′, (c.347G>C sense) 5′-AAGGAGCAGGACAGCCTTCGTGTGCCC-3′, (c.347G>C antisense) 5′-GGGCACACGAAGGCTGTCCTGCTCCTT-3′, (c.362A>T sense) 5′-GCCCCCATCGTGGACAAGGAGCAGGAC, and (c.362A>T antisense) 5′- GTCCTGCTCCTTGTCCACGATGGGGGC-3′. Sequencing confirmed that the respective mutations were incorporated, with no other alteration in the construct.

For lentivirus expression, human DNase II cDNA was transferred from pCMV6-RC209573 via SalI/NotI fusion to a modified version of pLenti6 (Invitrogen, ThermoFisher Scientific, Waltham, MA) with an elongation factor-1a (EF1a) promoter and encephalomyocarditis virus IRES sequence followed by an eGFP sequence and a blasticidin/SV40 promoter resistance cassette.

For CRISPR-Cas9-based editing of human DNase II, the gRNA GGCACTCATCAACAGCCCGG(AGG) was selected with the CRISPR design tool (Zhang Lab, MIT, crispr.mit.edu) and introduced into an EF1a-Cas9-U6-sgRNA expression plasmid via Gibson assembly^45^.

All DNase II degradation assays were performed using an empty pBluescript (SK-) bacterial vector.

### CRISPR-Cas9 genome editing

A CRISPR/Cas9/gRNA plasmid targeting DNASE2 was introduced into HEK293FT (Invitrogen) using *Trans*IT-LT1 Transfection Reagent (Mirus Bio, Madison, Wisconsin) according to the manufacturer’s instructions. Cells were then subjected to limited dilution and seeded as single-cell colonies. After expansion, DNase II-deficient clones were identified via Sanger sequencing. A clonal cell line with a heterozygous 7 bp deletion in Exon 2 was used for the experiments in this manuscript.

### Lentivirus expression and lentiviral transduction

Lentiviral particles were generated as previously described^46^. In brief, lentiviral transduction was performed using a second generation lentiviral vector pLenti (Invitrogen), which is a poor stimulator of the type 1 interferon response even during acute infection^47^ and which are not capable of replication. Viral supernatants were then subjected to ultracentrifugation using a Beckman Coulter SW32 rotor at 21000xg for 2 h. Viral pellets were then resuspended in DMEM and added to primary human fibroblasts at an MOI of 100. After 2 days, positive cells were selected using blasticidin.

### Purification and quantification of DNase II protein

Human DNase II constructs with a C-terminal FLAG sequence were introduced into DNase-II-deficient HEK293FT via calcium phosphate transfection. After 36 h, the cells were subjected to cytosolic lysis with a buffer containing 50 mM Tris HCl, pH 7.4, 150 mM NaCl, 50 mM EDTA, 1% TRITON X-100, and a protease inhibitor cocktail (cOmplete Protease Inhibitor Cocktail Tablets, Roche). Nuclei were removed via centrifugation, and the cytosolic supernatant was fractionated with ammonium sulfate and resuspended in 50 mM Tris HCl, pH 7.4, with 150 mM NaCl, 1 mM EDTA. The samples were then subjected to affinity immunoprecipitation with Anti-FLAG M2 Agarose Beads (Sigma) and eluted with FLAG Peptide (Sigma) according to the manufacturer’s instructions. The eluted fractions were precipitated with ammonium sulfate, resuspended and dialyzed with SpectraPor #4 tubing MWCO 12–14 kDa (Carl Roth, Karlsruhe, Germany) against 40 mM Tris-HCl 7.4, 25 mM EDTA, 100 mM NaCl, 1 mM DTT to remove FLAG Peptide and residual ammonium sulfate. Proteins were quantified using an in-house developed FLAG-based ELISA assay. In brief, high-binding plates were coated with the proteins of interest and defined amounts of purified IL-1b-FLAG were used as a standard. Plates were then blocked with 1% BSA overnight, and FLAG-tagged proteins were detected using HRP-coupled Anti-FLAG M2 antibody. These results were then verified via PAGE electrophoresis and immunoblotting with Anti-FLAG M2 antibody.

### Activity of purified DNase II protein and in cell lysates

Purified DNase II protein was combined with 1 µg plasmid DNA and incubated at pH 5.0 with 60 mM NaOAc and 50 mM EDTA for 1 h. Samples were then neutralized with a loading buffer containing 0.25% bromophenol blue, 25% glycerol, 80 mM Tris-HCl, pH 8, and 5 mM EDTA and subjected to electrophoresis in a 1% agarose gel containing 0.0001% SybrSafe (ThermoFisher, Waltham, MA, USA). DNA was visualized with a Licor^®^ Fc imaging system (Licor, Lincoln, Nebraska, USA).

DNase II activity in patient fibroblasts was measured using a modified version of the protocol from Howell^48^. In brief, 2 × 10^6^ fibroblasts were lysed in 100 µL lysis buffer, containing 1% Triton-X, 50 mM Tris-HCl, pH 7.4, 150 mM NaCl and 25 mM EDTA. Nuclei were removed by centrifugation, Purified plasmid DNA was diluted with assay buffer (60 mM sodium acetate, pH 5, 50 mM) to a final amount of 0.4 g per 21 µL reaction and aliquoted. Cellular supernatants were diluted 1:5 into the DNA solution and incubated at 37 °C as indicated. Samples were then neutralized with loading buffer, subjected to agarose gel electrophoresis and imaged as described above.

Determination of the rate of change of optical density was performed with a modified, microscaled version of the protocol from Kunitz^49^. In brief, 1 µg of plasmid DNA was incubated with the indicated concentrations and types of DNase II and 0.1 M NaOAc buffer (pH) in a total volume of 3 µL at 37 °C in a Biotek Take 3 microplate (Biotek, Winooski, Vermont, USA), and the absorbance at 260 nm was measured once per minute.

### Stimulation of primary human fibroblasts

Fibroblasts were seeded at 2 × 10^5^ cells per well in a 96-well plate. After 16 h, the cells were stimulated. cGAMP transfection was performed via digitonin permeabilization as previously described^50^ In brief, cells were permeabilized at 37 °C for 30 min with 5 µM 2′-3′cGAMP in 50 mM HEPES pH 7.0, 100 mM KCl, 3 mM MgCL_2_, 0.1 mM DTT, 85 mM Sucrose, 0.2% BSA, 1 mM ATP, 0.1 mM GTP and 10 µg/mL Digitonin. 5′ triphosphate dsRNA (5′ pppRNA) was created via in vitro transcription using a commercial T7 in vitro transcription kit with the template 5′- TTGTAATACGACTCACTATAGGGACGCTGACCCAGAAGATCTACTAGAAA TAGTAGATCTTCTGGGTCAGCGTCCC-3′ as in reference^51^. For stimulation with dsDNA and 5′ pppRNA, 200ng was transfected per well with lipofectamine 2000 (ThermoFisher) according to the manufacturer’s instructions.

### STAT phosphorylation assay staining

Peripheral blood mononuclear cells (PBMCs) were treated or untreated with ruxolitinib 1 µM for 2 h. Cells were fixed using Beckman Coulter PerFix Expose Fixation Buffer (10 min at room temperature) and then permeabilized using BC PerFix Expose Permeabilizing Buffer (5 min at 37 °C). Cells were stained with FITC-anti-STAT1 pY701 (BD Bioscience, cat: 612596, 4a, 1:5), PE-anti-STAT1 pY701 (BD Bioscience, cat: 612564, 4a, 1:5) or FITC-anti-STAT3 pY705 (BD Bioscience, cat: 612564, 4a, 1:5) and cell surface markers APC-CD3 (Miltenyi Biotec, cat: 160-109-462, REA613, 1:30) and APC-Alexa750-CD14 (Beckman Coulter, cat: B92421, RM052, 1:30)) for 1 h at room temperature protected from light. Flow cytometry analysis was performed on a Gallios Beckman Coulter flow cytometer. Results were analyzed using Kaluza software v1.3.

### Quantification of circulating protein biomarkers

Serum or plasma concentration of the following 65 immune monitoring biomarkers, APRIL, BAFF, BLC, CD30, CD40L, ENA-78, Eotaxin, Eotaxin-2, Eotaxin-3, FGF-2, Fractalkine, G-CSF, GM-CSF, Gro α, HGF, IFN-α, IFN-ɣ, IL-10, IL-12p70, IL-13, IL-15, IL-16, IL-17A, IL-18, IL-1 α, IL-1β, IL-2, IL-20, IL-21, IL-22, IL-23, IL-27, IL-2R, IL-3, IL-31, IL-4, IL-5, IL-6, IL-7, IL-8, IL-9, IP-10, I-TAC, LIF, MCP-1, MCP-2, MCP-3, M-CSF, MDC, MIF, MIG, MIP-1 α, MIP-1β, MIP-3α, MMP-1, NGF-β, SCF, SDF-1α, TNF-β, TNF-α, TNF-R2, TRAIL, TSLP, TWEAK, and VEGF-A, were assessed in samples from patients and healthy donors. Frozen sera or plasma were thawed and spun (1000 × *g*, 5 min) immediately before starting the experiment. Quantification was performed based on the Luminex xMAP technology (Luminex Corp., Austin, TX, USA), using the Immune Monitoring 65-Plex Human ProcartaPlex Panel (ThermoFischer Scientific, Waltham, MA, USA), according to the manufacturer’s instructions. Plates were analyzed on a Bioplex 200 analyzer (Biorad, Hercules, CA, USA). Concentrations were calculated from the raw data using the Five-Parameter Logistic (5PL) equation on the GraphPad Prism6 software (GraphPad Software, Inc., La Jolla, CA, USA). Heat maps were made with the R package ctc: Cluster and Tree Conversion and imaged by Java Treeview software.

### Single analyte ELISA assays

TNFα, IL-1β and IL-6 were measured by standardized ELISA assay (Invitrogen, KAC1751, KAC1211, KAC1261 respectively) in the diagnostic laboratory of the Hôpital Necker-Enfants Malades, and compared to a normal range of controls for each cytokine.

### Interferon alpha in serum and supernatant

As reported by Rodero et al.^26^, a Simoa interferon alpha (IFN-α) assay was developed using a Quanterix Homebrew Simoa assay and two autoantibodies specific for IFN-α isolated and cloned from two APS1/APECED patients recently described^24^. The 8H1 antibody clone was used as a capture antibody after coating on paramagnetic beads (0.3 mg/mL), and the 12H5 was biotinylated (biotin/Ab ratio = 30/1) and used as the detector. Recombinant IFN-α17/αI (PBL Assay Science) was used as a standard curve after cross-reactivity testing. The limits of detection (LOD) were calculated by the mean value of all blank runs + 3SDs and was 0.23 fg/mL.

### Cell sorting and culture for interferon alpha dosage

Peripheral blood mononuclear cells (PBMCs) were isolated from blood using lymphocyte separation medium. Just after isolation, PBMCs were labeled with CD3 Krome Orange (Beckman Coulter, cat: B00068, UCHT1, 1:30), CD19 PE-Cy7 (BD Bioscience, cat: 557835, SJ25C1, 1:100), CD56 FITC (Miltenyi Biotec cat: 130-100-746, REA196, 1:30) and CD14 APC-Alexa Fluor 750 (Beckman Coulter, cat: B92421, RM052, 1:30). PBMC subsets were isolated using a BD FACS Aria II. Purity of the cell sorting was verified for 9 individual donors, and was high for all populations (mean ± SD for CD3: 95.73% ± 3.02; B cells: 97.5% ± 2.3; NK: 97.9% ± 1.6; monocytes: 97.7% ± 2.6). After sorting, cells were cultured at 1,500,000 cells per mL in 96 wells plates with 100 µL of RPMI SVF10% for 48 h. Supernatant was isolated from non-adherent cells and debris by centrifugation (5 min, 10,000 × *g*), and stored at −80 °C until analyzed on the Simoa platform.

### Erythroid liquid culture

Erythroid cells were generated in vitro from peripheral blood circulating CD34^+^ cells obtained from the cord blood of healthy donors. Isolated CD34^+^ progenitors (Miltenyi Biotec CD34 Progenitor Cell Isolation Kit) were grown in the presence of 100 ng/mL IL-6, 10 ng/mL IL-3 and 100 ng/mL stem cell factor (SCF) for 7 days. On day 7, erythroid progenitors were switched to a second phase of culture allowing the differentiation and maturation of erythroblasts: thus, cells were cultured in the presence of 10 ng/mL IL-3, 50 ng/mL SCF and 0.2 U/mL erythropoietin (EPO) in IMDM (Gibco cell culture) supplemented with 15% BIT 9500 (Stem Cell Technologies), as previously described^52^. Cells were then cultured in the presence of 3 different doses of interferon alpha 2 (0, 100, and 1000 IU/L).

### Western blot analysis

Proteins were extracted from monocyte-derived macrophages enriched from PBMCs by plastic adhesion and from transfected HEKs using lysis buffer (RIPA) supplemented with 1% protease inhibitor and 1% phosphatase inhibitor (all from ThermoFisher Scientific, Waltham, Massachusetts, USA). Bolt LDS Sample Buffer (4×) (Novex Life Technologies) and Bolt Sample Reducing agent (10×) (Novex Life Technologies) were added to protein lysates, samples resolved on 8% Bolt Bis-Tris Plus gels (ThermoFisher Scientific, Waltham, Massachusetts, USA), and then transferred to nitrocellulose membrane (#IB23002, ThermoFisher Scientific, Waltham, Massachusetts, USA). Membranes were blocked by incubation with 3% non-fat milk powder in TBS and primary antibodies were incubated overnight. The appropriate Infrared Dye (IRDye)-conjugated secondary antibody (IRDye 680RD Goat Anti-Mouse IgG C/N 926-68070 or IRDye 800CW Goat Anti-Rabbit IgG C/N 926-32211; dilution 1:10,000; both from Li-Cor, Lincoln, Nebraska, USA) was incubated with the membrane before the detection of antibody binding by Licor Odyssey CLx system (Li-Cor, Lincoln, Nebraska, USA). Proteins were detected with polyclonal anti-DNASE2 (ab8119, dilution 1:500) and Vinculin (EPR8185, ab129002, dilution 1:50000) antibodies (both from Abcam, Cambridge, UK); monoclonal mouse mouse anti-β-Actin (Cell Signaling, Danvers, Massachusetts, USA, 8H10D10, 1:10,000) antibody. DNase II is glycosylated at multiple sites, giving a size at around 47 kDa^53^.

### Protein modeling: PDB reference

Experimental 3D structures of was based on a previously published structure prediction^17^ and analyzed using Chimera (http://www.cgl.ucsf.edu/chimera/).

### URLs

UCSC Human Genome Browser, http://genome.ucsc.edu/; Ensembl, http://www.ensembl.org/; dbSNP, http://www.ncbi.nlm.nih.gov/projects/SNP/; Exome Variant Server, NHLBI Exome Sequencing Project (ESP), http://snp.gs.washington.edu/EVS/; Exome Aggregation Consortium (ExAC), http://exac.broadinstitute.org (accessed January 2017); Clustal Omega, http://www.ebi.ac.uk/Tools/msa/clustalo/; Alamut, http://www.interactive-biosoftware.com/; GraphPad, http://www.graphpad.com/.

### Data availability

All relevant non-patient sensitive data are available from the authors. RNA-seq data are available at ArrayExpress E-MTAB-5735.

## Electronic supplementary material

Supplementary Information
