# Supplementary material for: Type I interferon-mediated autoinflammation due to DNase II deficiency
Source: Nat Commun. 2017 Dec 19;8:2176. doi: 10.1038/s41467-017-01932-3 (PMC5736616; doi:10.1038/s41467-017-01932-3)
Supplement: Supplementary file 1 — Supplementary Information [file 41467_2017_1932_MOESM1_ESM.pdf]

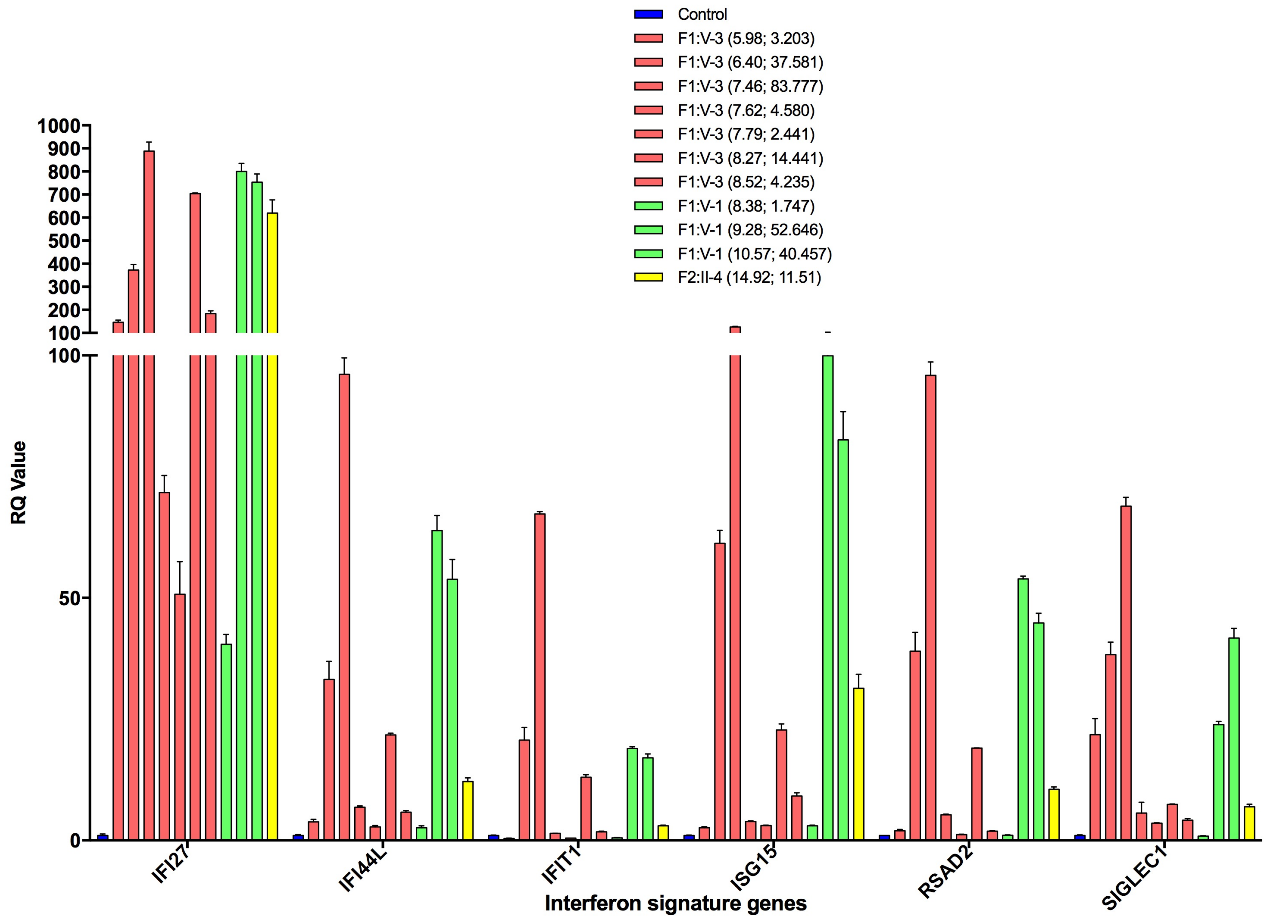


**Supplementary Figure 1. Quantitative RT-PCR (qPCR) of a panel of six interferon stimulated genes (ISGs) in *DNASE2* mutation–positive probands and controls**. Bar graphs show relative quantification (RQ) values for the expression of *IFI27*, *IFI44L*, *IFIT1*, *ISG15*, *RSAD2* and *SIGLEC1* measured in whole blood of patients, compared to the combined results of 29 healthy controls. The RQ value is equal to $2^{-\Delta\Delta Ct}$, with error bars of $-\Delta\Delta Ct$ ± SD (i.e., normalized fold change relative to a calibrator) shown. Decimalized age is given in brackets, followed by the interferon score - calculated from the median fold change in RQ value for the panel of six ISGs. The mean interferon score of the controls + 2 SD above the mean was calculated. + 2 SD is chosen as a conservative approach to the analysis of the data, with scores higher than this value (2·466) designated as positive.

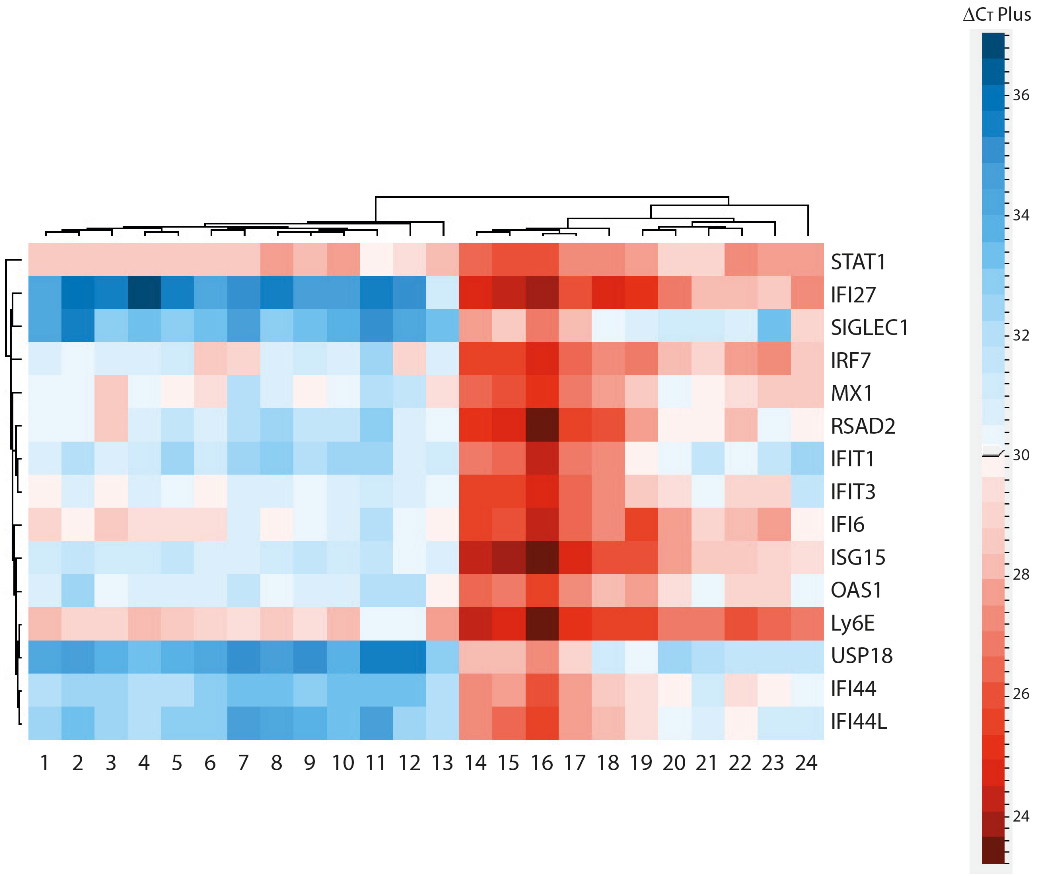


**Supplementary Figure 2. Heat map showing the expression of an extended panel of 15 interferon stimulated genes (ISGs) in patient and control samples**. Distances between samples and probes were calculated for hierarchical clustering based on the $\Delta C_T$ values using Pearson's Correlation and Average Linkage. Scale represents $\Delta C_T$ + global control mean ($\Delta C_T$ value of HPRT1 and 18S). Assay Centric view is shown (the middle expression level is set as the mean of all the $\Delta C_T$ values for each probe). Lanes 1 to 13 are control samples (M10, F6, F12, M2, M13, F40, M30, M22, F23, M4, F22, F37, M1 where M and F = male and female respectively, and the number is the age in years at time of sampling). Lane 14 F1:V-1 (10.57; 40.457); Lane 15 F1:V-1 (9.28; 52.646); Lane 16 F1:V-3 (7.46; 83.777); Lane 17 F1:V-3 (6.40; 37.581); Lane 18 F1:V-3 (8.27; 14.441); Lane 19 F2:II-4 (14.92; 11.51); Lane 20 F1:V-3 (8.52; 4.235); Lane 21 F1:V-3 (7.79; 2.441); Lane 22 F1:V-3 (7.62; 4.58); Lane 23 F1:V-1 (8.38; 1.747); Lane 24 F1:V-3 (5.98; 3.203)(where numbers in brackets before and after the semicolon are the decimalized age and the interferon score, see legend to Figure S1 above, respectively).

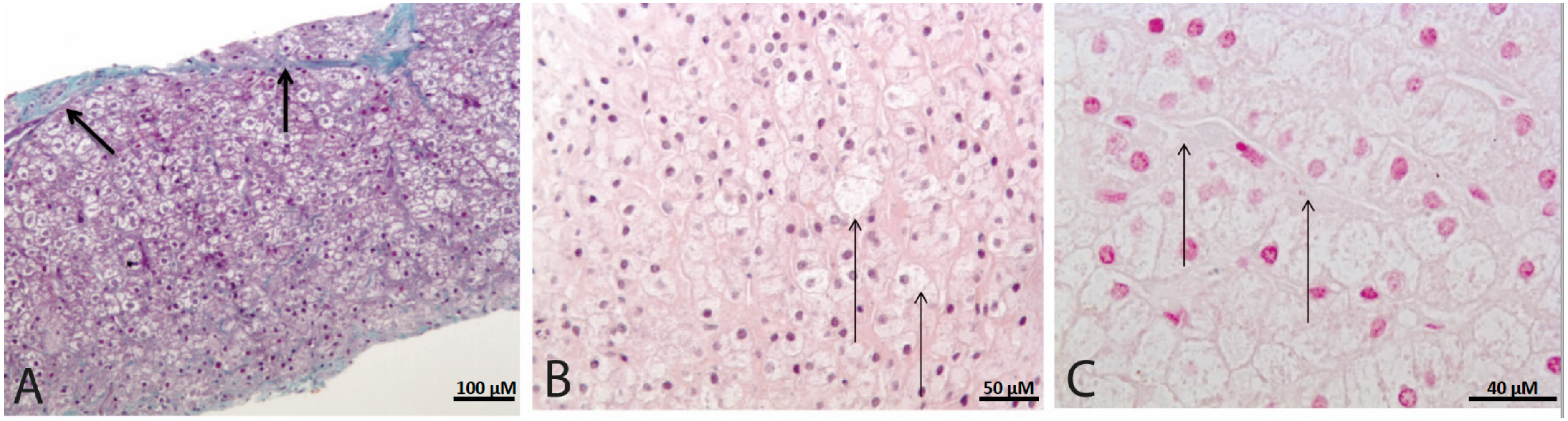


**Supplementary Figure 3. Findings on biopsy of the liver of F1:V-3 at age 8 years**. Arrows demonstrate bridging fibrosis (A: trichrome staining; x10), ballooning of hepatocytes (B: HES staining; x20) and microvacuoles with debris in the cytoplasm of enlarged Kupffer cells (C: Perls' staining; x40).

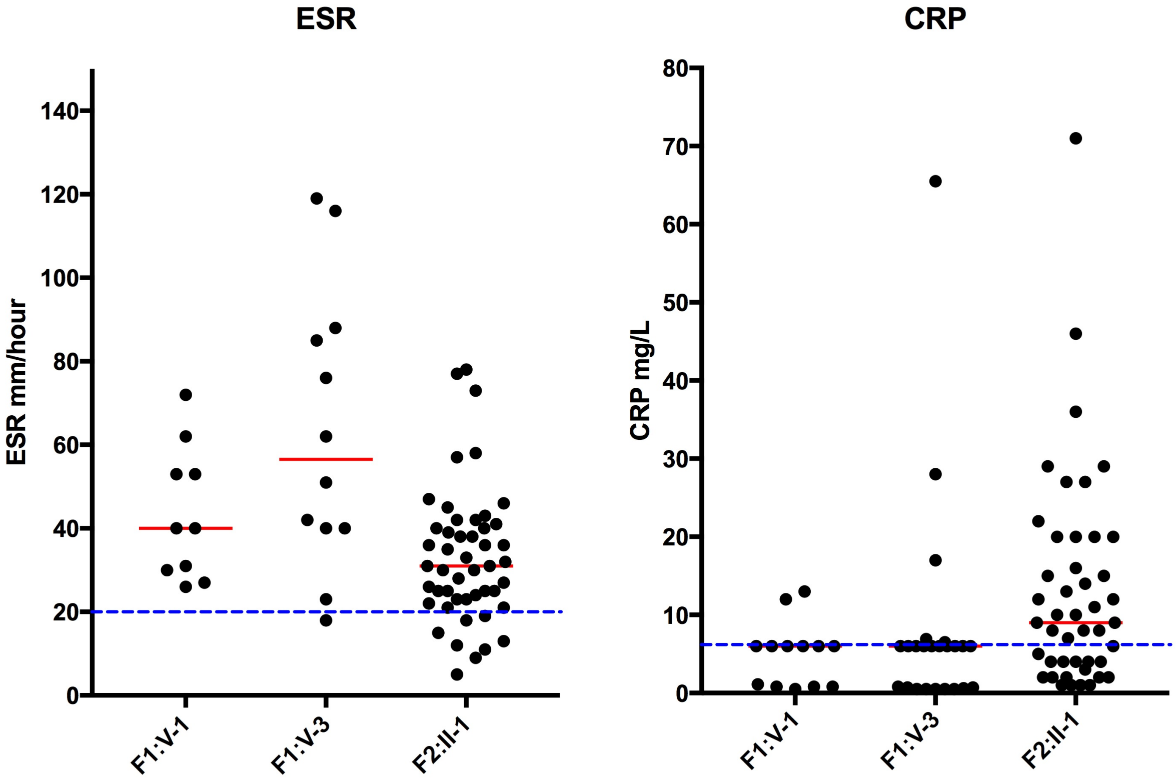


**Supplementary Figure 4. Erythrocyte sedimentation rare (ESR) and C-reactive protein (CRP) data measured in the three affected individuals with biallelic mutations in *DNASE2*.** Red line indicates median value; dotted blue line indicates the upper limit of the normal range (20 mml/hour and 6 mg/l for ESR and CRP respectively).

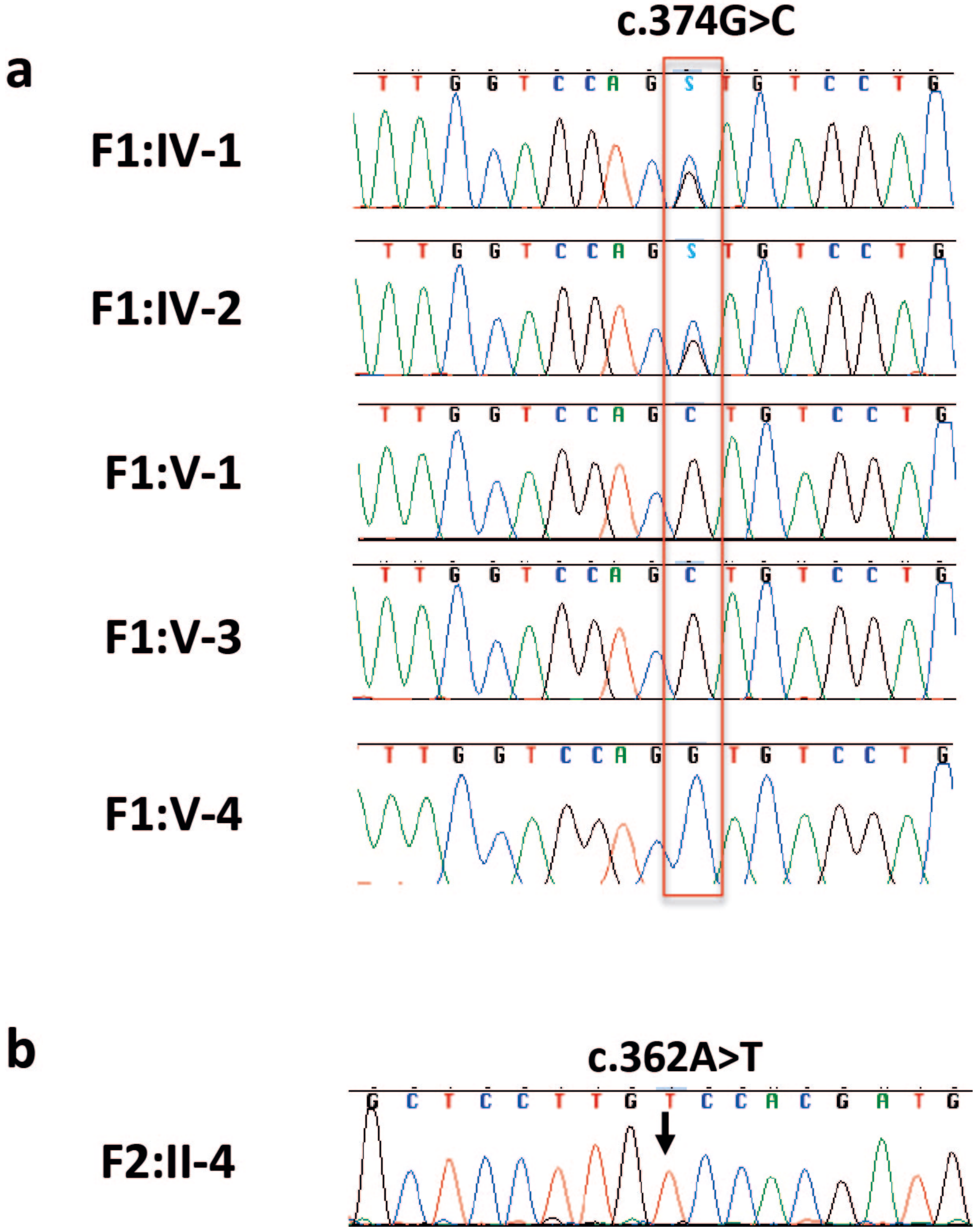


**Supplementary Figure 5. Sanger sequencing of mutations. a.** Red box highlights c.374G>C change in family F1. **b**. Arrow highlights c.362A>T change in family F2.

```
Human           --------MIPLLL-AA--LLCVPAGALTCYGDSGQPVDWFVVYKLPALRGSGE--AAQR
Chimp           --------MIPLLL-AA--LLCVPAGALTCYGDSGQPVDWFVVYKLPALRGSGE--AAQR
Gibbon          --------MIPLLL-TA--LLCVPAGALTCYGDSGQPVDWFVVYKLPALRGSGE--AAQR
Rat             -----MAAPSSLLL-AA--LLWVPAEALSCYGDSGRPVDWFVVYKLPANSGSGD--KPWK
Mouse           -----MATLRSLLL-AA--LLWVPAEALSCYGDSGQPVDWFVVYKLPAHSGSRD--TP-K
Dog             -----MATLSPLLL-AA--LLWVPVRTLTCYGDSGQPVDWFVVYKLPAQSGPGD--AAQR
Xenopus         -------MLLRLLMHLI--LPLGSFAATSCYGDHGKQVDWFIVYKLPQLRNKGE--E--A
Tetraodon       -------MLLFLLVFAL--PVEVNSSPISCYNDQGAAVDWFYLYKLPKEDRM-E--PQEE
Zebrafish       -------MFLHVLLLLH--ASRGAFSSISCYNDQGKPVDWYYLYKLPHEHH--T--PLEE
Drosophila      -----MRSLCFVLLFVWFFYQNEAKSKVSCKDEAGNDVDWWHLYKLPKHYQHNDLGKDTS
C.elegans       MGLSPAAVLIFLLL-----GVSQTYAAFSCKDQSGNDVDWFAVYKMPIEKDDGSVTGLAG
                           :*:              :* .: *  ***: :**:*

Human           GLQYKYLD-ESSGGWRDGRALINSPEGAVGRSLQPLYRSN-TSQLAFLLYNDQPPQPSK-
Chimp           GLQYKYLD-ESSGGWRDGRGLINSPERAVGRSLQPLYRSN-TSQLAFLLYNDQPPQPSK-
Gibbon          GLQYKYLD-ESSGGWRDGRGLINSPEGAVGRSLQPLYRSN-TSQLAFLLYNDQPPQPSK-
Rat             GLMYKYMD-QNSEGWQDGVGHIDSKDGAVGLTLQPLYQKN-SSQLAFLLYNDQPPKSSS-
Mouse           GLTYKYMD-QNSDGWQDGVGYINSSEGAVGRSLQPLYRKN-SSQLAFLLYNDQPPKSSS-
Dog             GLRYKYMD-KDSGGWRDGAGSINSSAGAVGRSLLPLYQ-N-ASQLAFLLYNDQPPKSSG-
Xenopus         GMTYVYQD-SSSGGWVPGATLMNSTDSAVGQTVSQLYKAFGMKDVAYILYNDEPANISS-
Tetraodon       GDRYLLLE-KGSEEWSNGQGTVNDTTGALGRTVGQLYSQGKNTERAYILYNDQKPTVDLT
Zebrafish       GLKYLFMD-VESEGWTDGTTLVNDTQSAVGRTVGPLYEGGD---VGYILYNDQPPKQKQ-
Drosophila      GLKYLYVTSQNYDTWQMSGKFISDPLSLPAQTLNPLNDDP--SHTLLAAYNDQQPNGTV-
C.elegans       GVAWYYVDVNKKGTLTPSAKTLDDNDQAIAYTLQQYYDKQNDKTIFHVMYNDEPWGSKST
                *  :             .   :..     . ::                ***:

                              G116A D121V
Human           --------------------AQDS-SMRGHTKGVLLLDHDGGFWLVHSVPNFPPPASSAA
Chimp           --------------------AQDS-SMRGHTKGVLLLDHDGGFWLVHSVPNFPPPASSAA
Gibbon          --------------------AQDS-SMRGHTKGVLLFDHDGGFWLIHSVPNFPPPASSAA
Rat             --------------------AQDS-SSRGHTKGVLLLDQEGGFWLVHSVPRFPSPASSGA
Mouse           --------------------ARDS-TGHGHTKGVLLLDQEGGFWLVHSVPRFPPPASSGA
Dog             --------------------SQDFVAIRGHTKGVLLLDQEGGFWLVHSVPQFPAPASSGA
Xenopus         --------------------S---GSDRGHTKGMLLLDKKQGFWLVHSTPRFPPPAD-QS
Tetraodon       DR-----------------RVHMSGSSRGHTKGVVLLDKHQGFWLVHSTPHFPPAQKEGQ
Zebrafish       --------------------VEGASRSCGHTKGVVVFDKEQGFWLVHSTPHFPPPKSEGQ
Drosophila      ------------------------FSSGGHAKGVVASDGETAIWIVHSVPKFPTI---PD
C.elegans       SGIKLEEILSNRVYSNYTHEDDSTSTAFGHTKGTIFFDGTSGVWLVHSVPLFPNP---TK
                            **:**  :  *   ..*::**.* **

Human           YSWPHSACTYGQTLLCVSFPFAQFSKM--GKQLTYTYPWVYNYQLEG--IFAQEFPDLEN
Chimp           YSWPHSACTYGQTLLCVSFPFAQFSKI--GKQLTYTYPWVYNYQLEG--IFAQEFPDLEN
Gibbon          YSWPHSACTYGQTLLCVSFPFAQFSKI--GKQLTYTYPQVYNYQLEG--IFAQEFPNLEN
Rat             YSWPPNARTYGQTLLCVSLPFSQFPGI--GKQLTYTYPLVYDHKLEG--IFAQKLPDLEE
Mouse           YTWPPNAQTFGQTLLCVSLPFTQFARI--GKQLTYTYPLVYDHKLEG--FFAQKLPDLET
Dog             YSWPSNAHTYGQTLLCVSFPLAQFWKI--GRQLTYTYPLVYDHKLDA--DFAQKVPYLKD
Xenopus         YDWPLSAHRNGQSFLCVTYPYKQFGDI--GQQLLYNTILPYDSSIPE--DFSVDFPELKT
Tetraodon       YYYPGSGLINGQNFICVTYPLERFQTIDTGEQLQINQPSVYDCDVPQ--SLASLVPALAA
Zebrafish       FSYPTSGISNGQNFICVTYPFERFQTI--GEQLKINQPHIFDCSIPG--SLATAVPAMAQ
Drosophila      YSYPTSGEQYAQSMLCVTLKGEDLEKV--GQILVYNEPHFYYQRNPLATRSDELFPSLER
C.elegans       YEYPVSGHDYGQTMLCMTFKYAQLKSI--GTQLFFNRPNIYSSNLPT--NMAADNADLAK
                : :* ..   .*.::*::     :  :  *  *  .    :                :

Human           VVKGHH-------VSQEPWNSSITLTSQAGAVFQSFAKFSKFGDDLYSGWLAAALGTNLQ
Chimp           VVKGHH-------VSQEPWNSSITLTSQAGALFQSFAKFSKFGDDLYSGWLAAALGTNLQ
Gibbon          VVKGHH-------VSHEPWNSSITLTSQAGAVFQSFAKFSKFGDDLYSGWLAAALGTNLQ
Rat             VTKGHH-------VLREPWNSSVILTSRAGTTFQSFAKFGKFGDDLYSGWLAAALGTNLQ
Mouse           VIKNQH-------VLHEPWNSSVILTSQAGATFQSFAKFGKFGDDLYSGWLAEALGTNLQ
Dog             VVKGHH-------VLHSPWNSSVTLTSKAGDTFQSFAKYGKFGDDLYSGWLAEALGSNLQ
Xenopus         AAEKGA-------VTQPPWNRQVVLTSVGGKQFTSFSKHAHFSDDLYSGWVSQVLKSHLF
Tetraodon       VCGGKATYGRASPQIQRVANRSVTLTSAGGTNFISFAKGASFKNDLYHAWVAPALQSDLL
Zebrafish       LCKHTL--DRWDNTSSSPSNRSISLLSLAKTEFISFAKGASFANDLYHSWVAPTLQSNLL
Drosophila      ALHGQW-------RTESPFQKDLEVRSLDGKKFRLFGKSGRANVELYADVVAPTLDVSLF
C.elegans       AIAGQY-------QKGQPFQSVIELETMAGYSFTNFAKSKEFNADLYDTLVAPTLKTDLV
                                   :  : : :     *  *.*      :**   :: .*    *
```

```
Human           VQFWHKTVGILPSNCSDIWQVLNVNQIAFPGPAGPSFNSTEDHSKWCVSP----------
Chimp           VQFWHKTVGILPSNCSDIWQVLNVNQIAFPGPAGPSFNSTEDHSKWCVSP----------
Gibbon          VQFWHKTVGILPSNCSDIWQVLNVNQIAFPGPAGPSFNSTEDHSKWCVSP----------
Rat             VQFWPNSPGILPSNCSGTHKILDVTETGFPGPSGPTFNATEDHSKWCVAP----------
Mouse           VQFWQNSPGILPSNCSGAYQVLDVTQTGFPGPSRLTFSATEDHSKWCVAP----------
Dog             VQFWQNSHGILPSNCSRVQHVLDVTQIAFPGPAGPAFSAREDHSKWCVAP----------
Xenopus         VQFWQNSRGVLPSNCSLPFHTYNIMEIDIS--CAYSFSTHNDHSKWCVTD----------
Tetraodon       VQFWVRSTGVLPSDCSLGWKVLDVTRINPG--KTSEFKASQDHSKWAVSTKAAG-----S
Zebrafish       VQFWRRSTGILPSDCSPNWEVLNIDLISPG--QKVTFKATEDHSKWAVSS---S-----S
Drosophila      VEAWRDGAGNLPNSCDKSDKVLNVESISNPE-LSVDFKTTQDHSKWAVSRPTGILIYHWR
C.elegans       VETWRRGSE-IPLDCKLTYHANDALSIHVG--STTAFSYTKDHSKMAHSAD---------
                *: *      :* .*.   .  :             *.  :**** . :

Human           --KGPWTCVGDMNRNQGEEQRGGGTLCAQLPALWKAFQPLVKNYQPCNGMARKPSRAYKI
Chimp           --KGPWTCVGDMNRNQGEEQRGGGTLCAQLPALWKAFQPLVKNYQPCNGMARKPSRAYKI
Gibbon          --KGPWTCVGDMNRNQGEEQRGGGTLCAQLPALWKAFQPLVKNYQPCNGMARKPSRAYKS
Rat             --EGPWVCVGDMNRNKRETHRGGGTLCTQVPALWKAFRSLVKACKPC-------------
Mouse           --QGPWACVGDMNRNKAETHRGGGTVCTQLPSFWKAFQSLVKDWKPCIEGS---------
Dog             --EGPWACVGDMNRNLGEEHRGGGTLCAQLPALWKAFQPLVKAWKPCGENRTSLQEIQQS
Xenopus         --GAGWACVGDMNRDVKEERRGGGTVCVNDPNVWKSFRSLVSSYNNCTGPVSYSRV----
Tetraodon       GAGGGWVCVGDINRNMAEEKRGGGTVCLRDPVVWKAYRTAVVDYEDCGRDKKSPICVLQL
Zebrafish       GTSGGWVCVGDINRDEAEEKRGGGTVCRRDAVVWKAYRSAALQCESCSGEVQTCEQAQRF
Drosophila      VGGGDWICVGDINRQEGQLHRGGGTVCHKSARVSNLYRQLVTNYDKCAQQE---------
C.elegans       -MTKPWVCIGDINRMTSQYVRGGGTTCISSSFLWKAYSVIATQ-NNCA------------
                     * *:**:**   :  ***** *     . : :   .   . *

Human           -
Chimp           -
Gibbon          -
Rat             -
Mouse           -
Dog             I
Xenopus         -
Tetraodon       -
Zebrafish       R
Drosophila      -
C.elegans       -
```

**Supplementary Figure 6. CLUSTAL Omega alignment of DNase II homologs**. DNase II homologs were identified on Ensembl and aligned using CLUSTAL Omega. Amino acids altered by *DNASE2* mutations are highlighted by a red box. *H.Sapiens*, *Homo sapiens* (ENSG00000105612); *P. troglodytes*, *Pan troglodytes* (ENSPTRG00000010546); *N .leucogenys*, *Nomascus leucogenys* (ENSNLEG00000012861); *R .Norvegicus*, *Rattus norvegicus* (ENSRNOG00000023830); *M. Musculus*, *Mus musculus* (ENSMUSG00000003812); *C. Familiaris*, *Canis lupus familiaris* (ENSCAFG00000017115); *X. Tropicalis*, *Xenopus tropicalis* (ENSXETG00000007923); *T.nigroviridis*, *Tetraodon nigroviridis* (ENSTNIG00000003900); *D. Rerio*, *Danio rerio* (ENSDARG00000073893); *D.melanogaster*, *Drosophila melanogaster* (FBgn0000477); *C. elegans*, *Caenorhabditis elegans* nuc-1 (WBGene00003828). Homology to the human DNASE II reference sequence (ENSG00000105612): human – *P. troglodytes*, 98.89%; human – *N. leucogenys*, 97.5%; human – *R. norvegicus*, 72%; human – *M. musculus*, 70.8%; human – *C. familiaris*, 70.3%; human – *X. tropicalis*, 46.02%; human - *T. nigroviridis*, 39.11%; human – *D. rerio*, 40.33%; human – *D. melanogaster*, 31.15%; human - *C. elegans*, 30.93%.

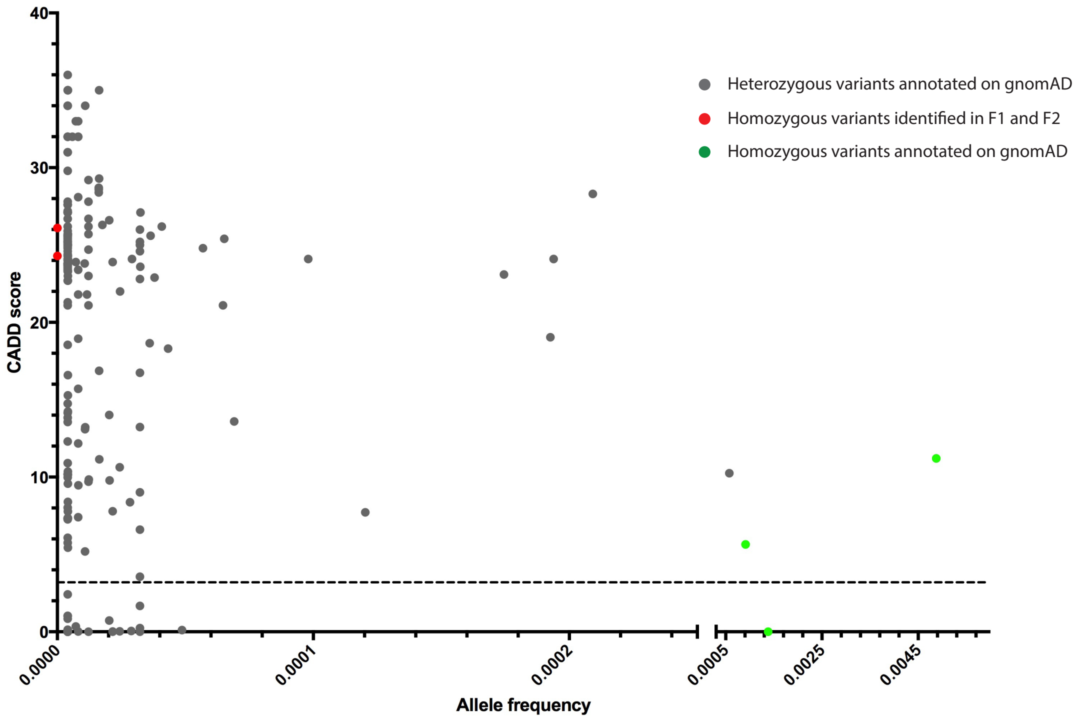


**Supplementary Figure 7. Plot of patient mutations and all *DNASE2* variants recorded in gnomAD**. All heterozygous (grey) and homozygous (green) variants recorded in gnomAD (http://gnomad.broadinstitute.org) for the gene *DNASE2* plotted according to allele frequency and combined annotation-dependent depletion (CADD: http://cadd.gs.washington.edu) score with the mutation significance cutoff (MSC: http://lab.rockefeller.edu/casanova/MSC)[1] given by the dotted line. Note that there are only three homozygous variants recorded in gnomAD, all at much high frequency and with lower CADD scores than the two homozygous variants identified in F1 and F2 in this study (red).

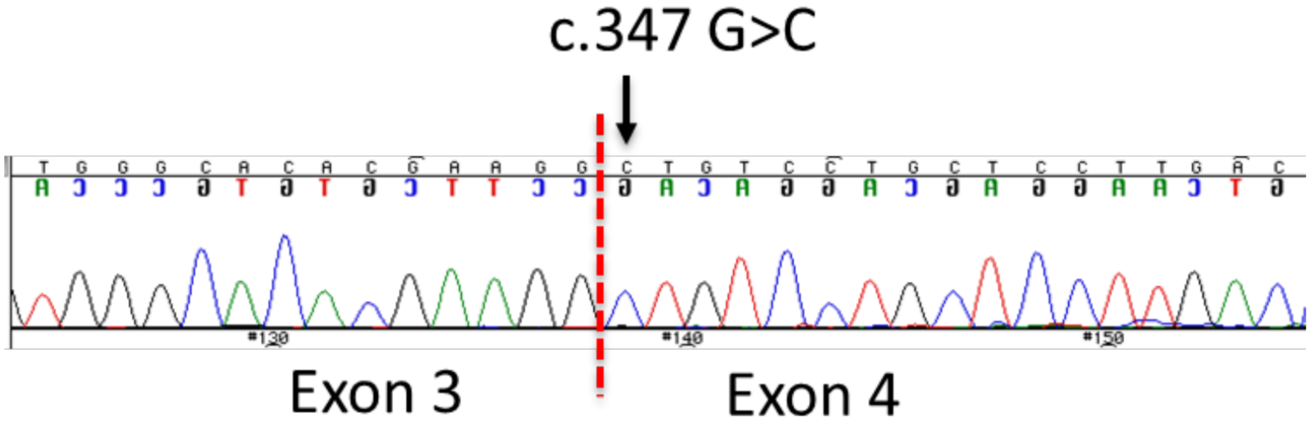


**Supplementary Figure 8**. **cDNA sequencing in F1:V-1**. Sanger sequencing of the faint band of wild-type size obtained on qPCR of cDNA recorded in Figure 2d from F1:V-1 indicating the presence of full length transcript including the c.347G>C (p.Gly116Ala) mutation.

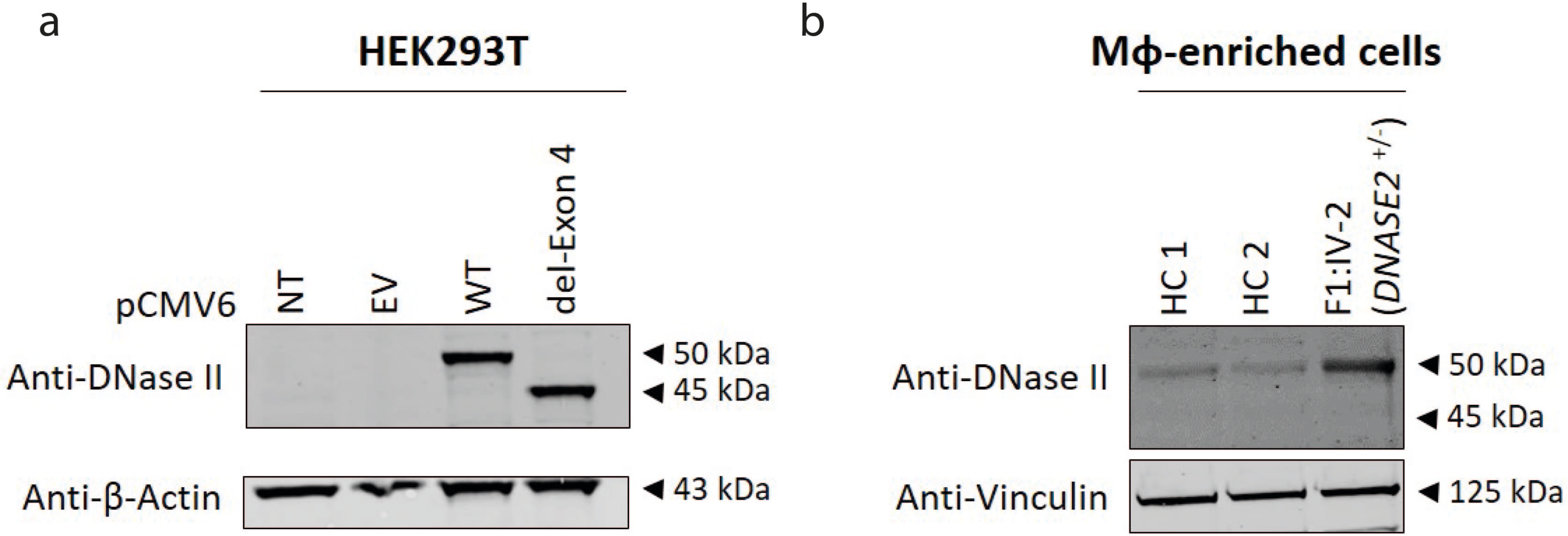


**Supplementary Figure 9**. **Overexpression of protein constructs in HEK293T cells**. **a**. Western blot of HEK293T cells transfected with a wild-type (WT) DNase II construct, and with a plasmid containing a deletion of exon 4 of DNase II (del-Exon 4). **b**. The results of an analysis of macrophage-enriched cells (MΦ) from two healthy controls (HC) and from F1:IV-2 (the mother of the two affected children in family F1). NT = Non-transfected; EV = Empty vector.

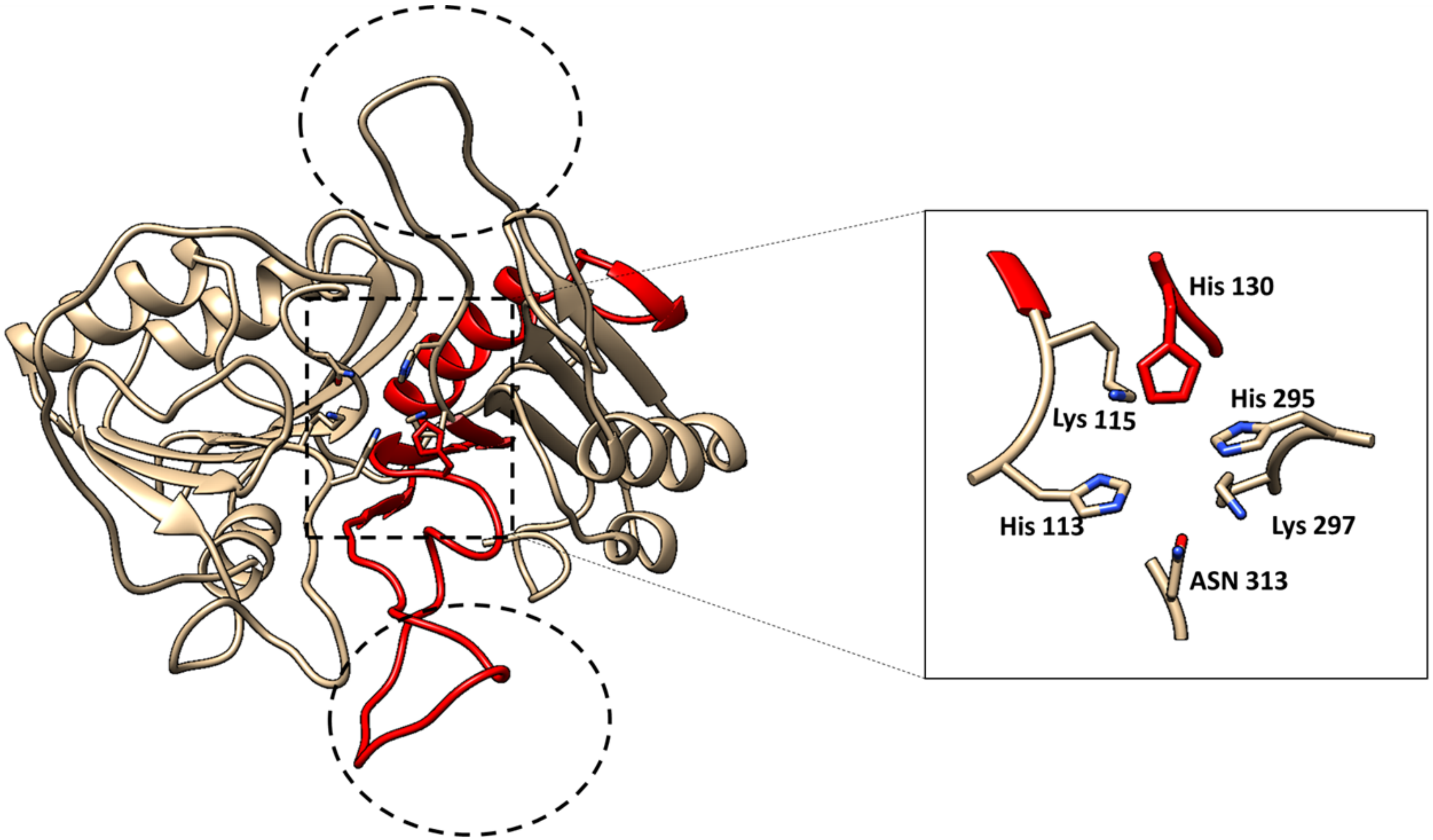


**Supplementary Figure 10**. **A model of the structure of DNase II**. The part of the protein encoded by exon 4 is depicted in red, and includes the catalytic core (dotted square), and a DNA binding domain (lower dotted circle; with a separate DNA binding domain indicated by the upper dotted circle).

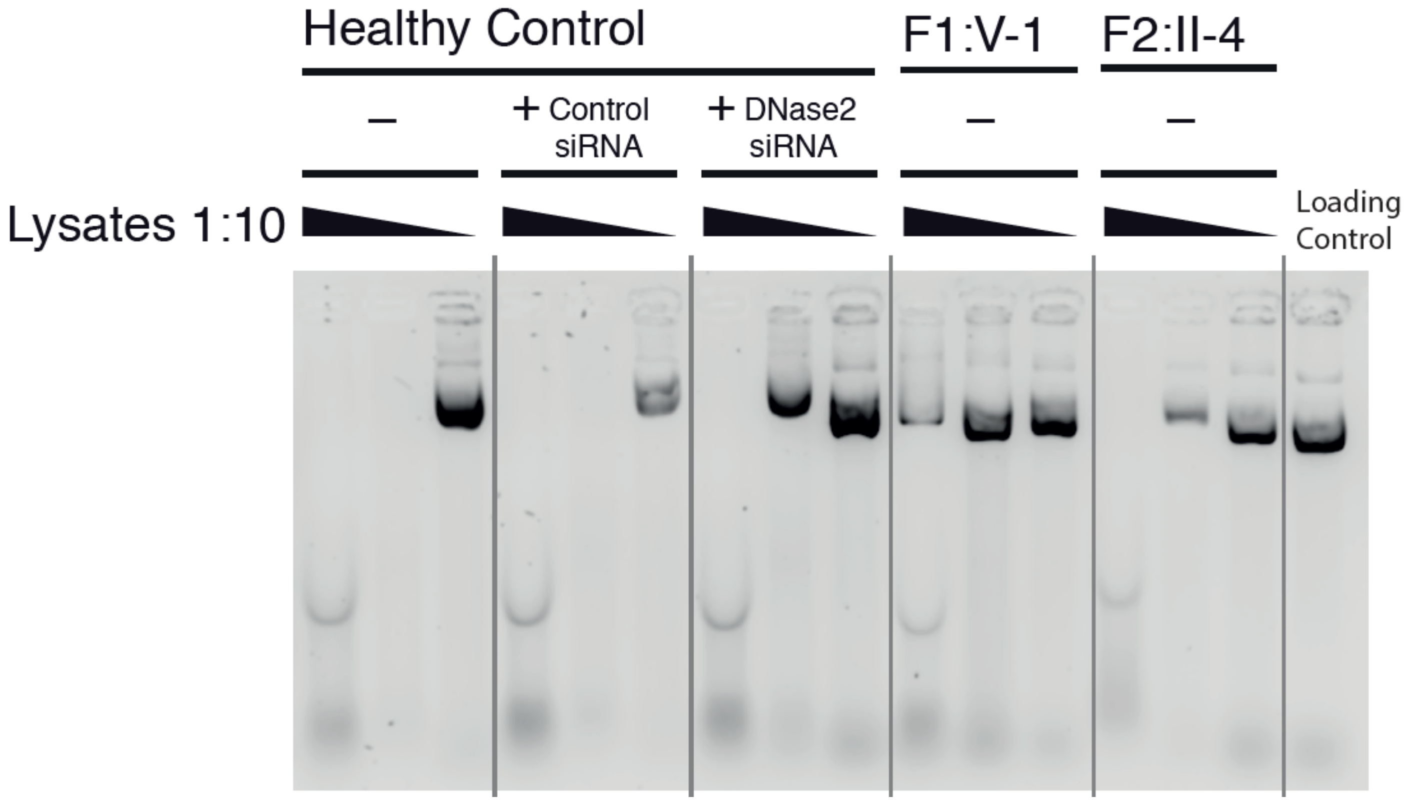


**Supplementary Figure 11. DNASE2 siRNA knock-down.** siRNA knock-down of *DNASE2* in fibroblasts results in a loss of nuclease activity against circularised plasmid DNA similar to that observed in patient cells.

a

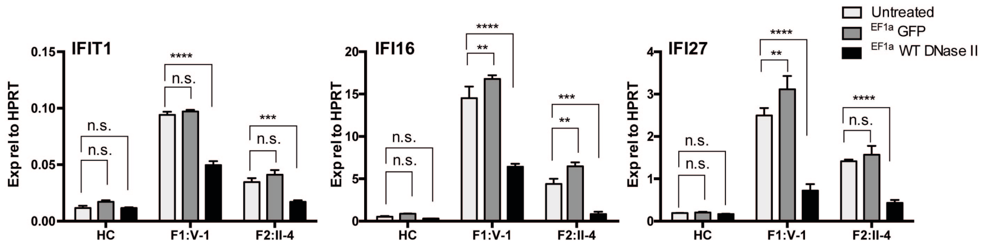


b

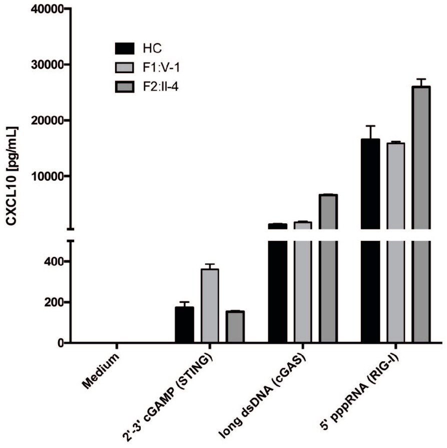


**Supplementary Figure 12**. **Interferon stimulated gene (ISG) expression in patient fibroblasts**. **a.** Compared to healthy controls (HC), unstimulated fibroblasts from F1:V-1 and F2:II-4 express increased levels of the ISGs *IFIT1*, *IFI16* and *IFI27*, which could be reduced by transfection of a wild-type (WT) DNase II second generation lentiviral vector pLenti (Invitrogen) construct, but not an empty GFP construct. HRPT expression was used for normalization. Data shown are mean +/- SD (n=2), and are representative of 3 independent experiments. **b**. To confirm the presence of the relevant functional pathways, fibroblasts from F1:V-1, F2:II-4 and a healthy control (HC) were shown to be similarly responsive to agonists of STING, cGAS and RIG-I (right-hand panel). 5' triphosphate (ppp) ds RNA is an agonist of RIG-I. Long dsDNA is an agonist of cGAS, and 2'-3' cGAMP is a direct agonist of STING. CXCL10 levels were measured 16h after stimulation by ELISA. Data represent mean ± SD (**p < 0.01, ***p < 0.001, ****p < 0.0001, two-way ANOVA) and are representative of two independent experiments.

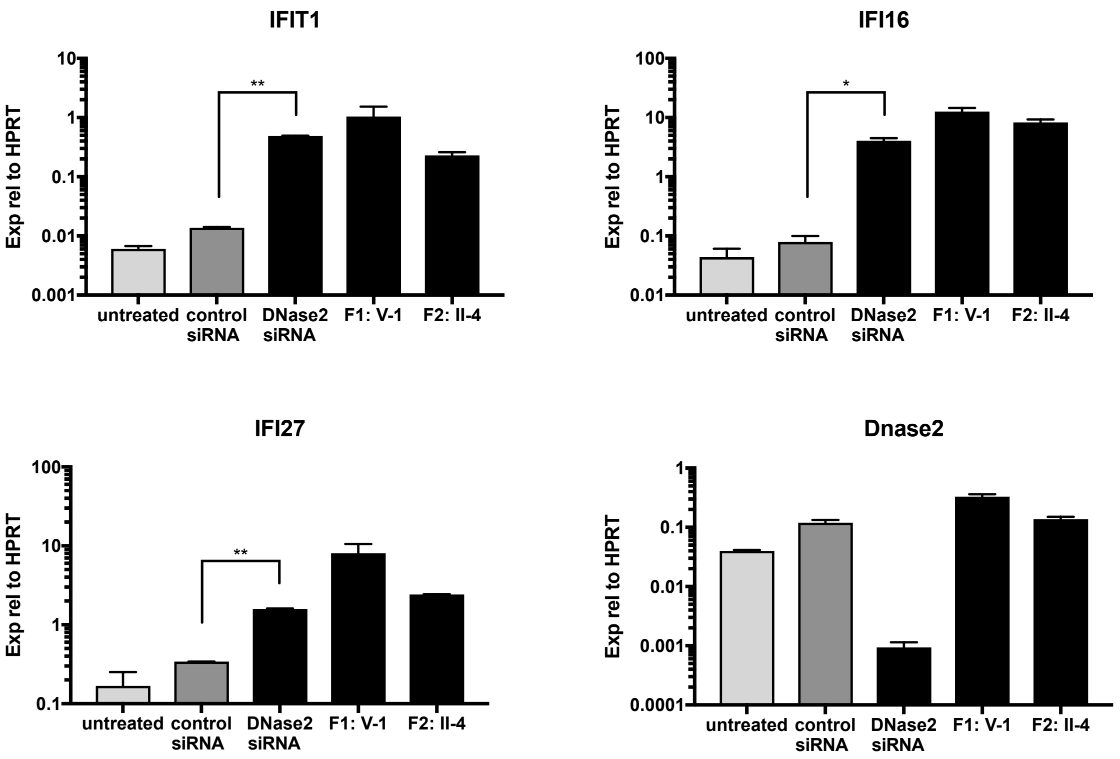


**Supplementary Figure 13. *DNASE2* siRNA knock-down**. siRNA knock-down of *DNASE2* in fibroblasts results in increased expression of interferon stimulated genes (ISGs) similar to that observed in patient cells. Healthy control (HC) fibroblasts were transfected twice in a 48 hour period with pooled siRNA targeting *DNASE2* or a scrambled control. After 24h cells were harvested for RNA extraction and RT-PCR analysis. Untreated HC and patient fibroblasts (F1:V-1 F2:II-4) were included for comparison. RT-PCR was performed for the ISGs IFIT1, IFI16 and IFI27 and for *DNASE2* to measure knockdown efficiency. HRPT expression was used for normalization. Data shown are mean +/- SD (n=2), and are representative of 2 independent experiments.

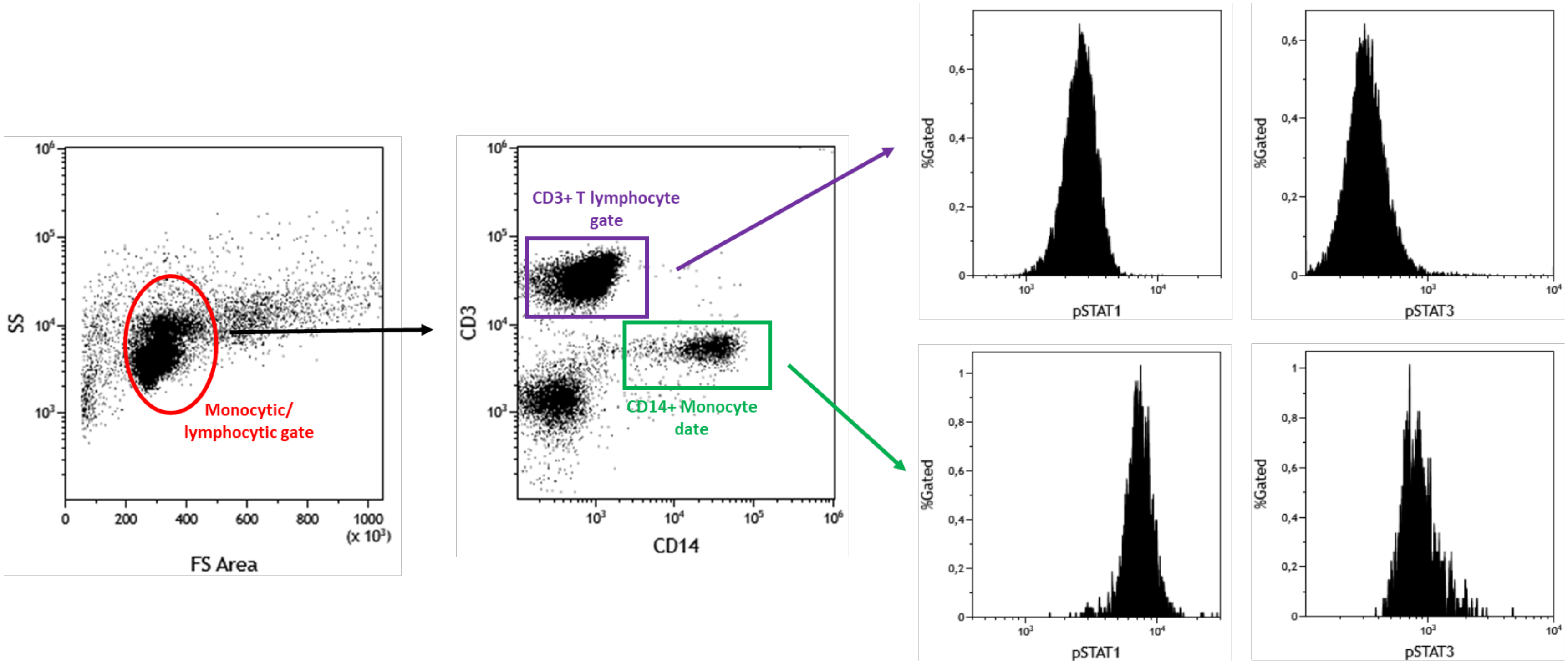


**Supplementary Figure 14. Gating strategies for STAT1 and STAT3 phosphorylation status.** a) Phosphorylation status was measured in CD3+ lymphocytes and CD14+ monocytes.

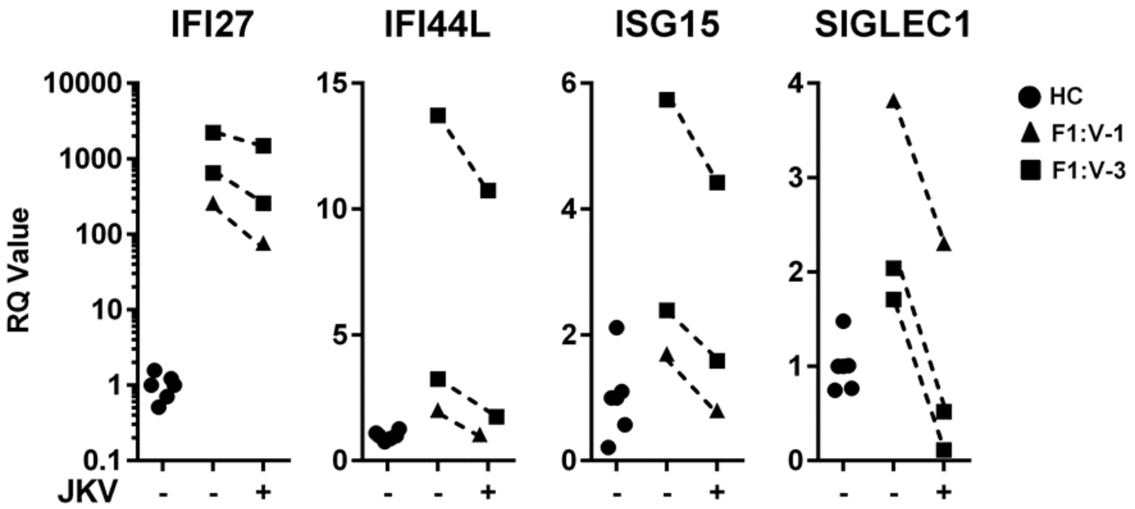


**Supplementary Figure 15**. **Interferon stimulated gene (ISG) production in monocyte-enriched cell fractions before and after treatment with ruxolitinib**. Increased expression of selected ISGs in cell fractions enriched for monocytes from F1:V-1 and F1:V-3 with or without the JAK1/2 inhibitor ruxolitinib.

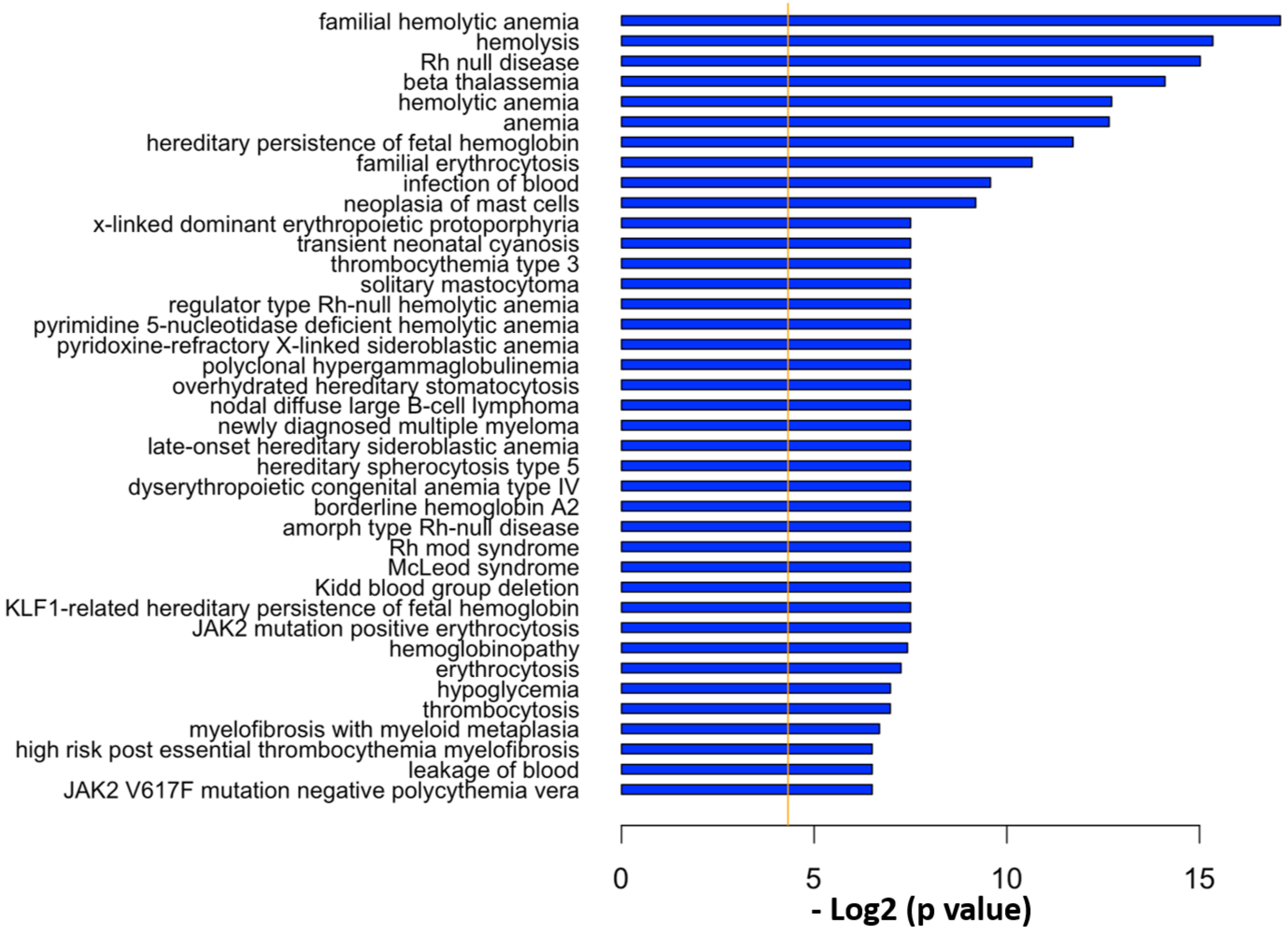


**Supplementary Figure 16. Ingenuity Pathway Analysis (IPA) of RNAseq data.** Histogram of hematological functions identified as significantly enriched according to Ingenuity Pathway Analysis (IPA) of RNAseq analysis selected according to the following criteria: Control *versus* DNase II fold change > 2 or < -2, (Adj p<0.05) and STING *versus* Controls (Adj p>0.05) and DNase II *versus* STING (Adj p<0.05).

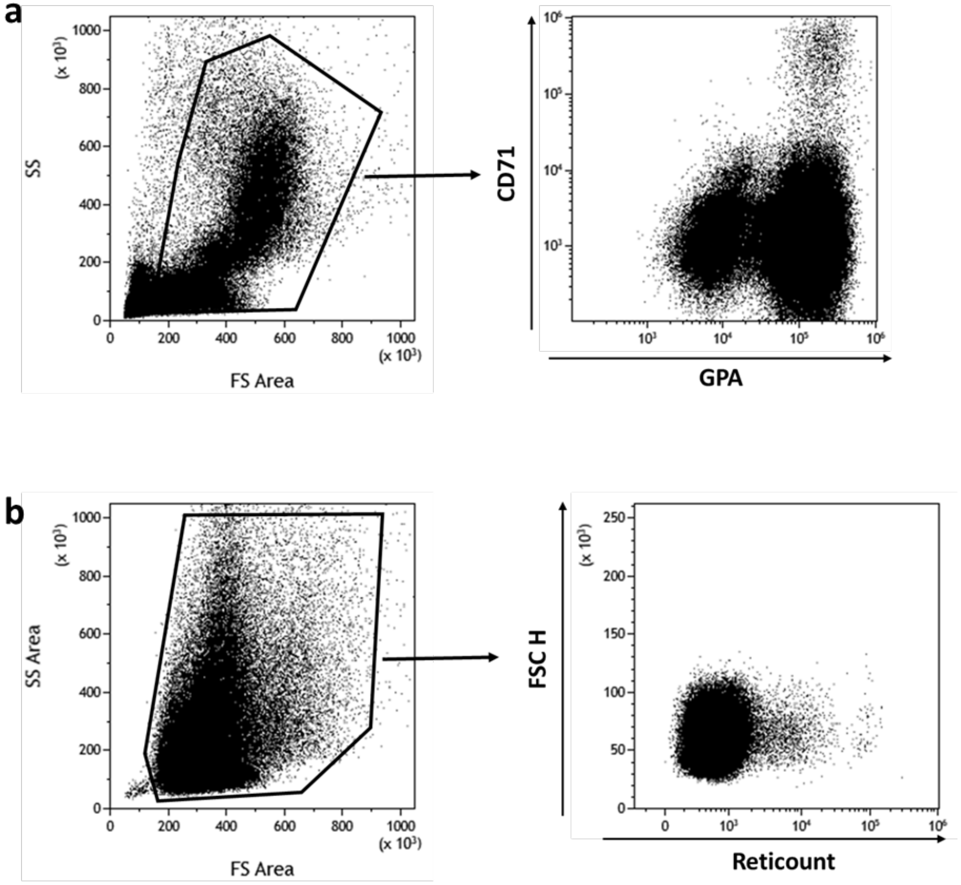


**Supplementary Figure 17. Gating strategies for hematopoietic studies.** a) Circulating erythroblasts are identified based on CD71 and GPA expression. b) Circulating reticulocytes are identified based on “reticount” marker levels.

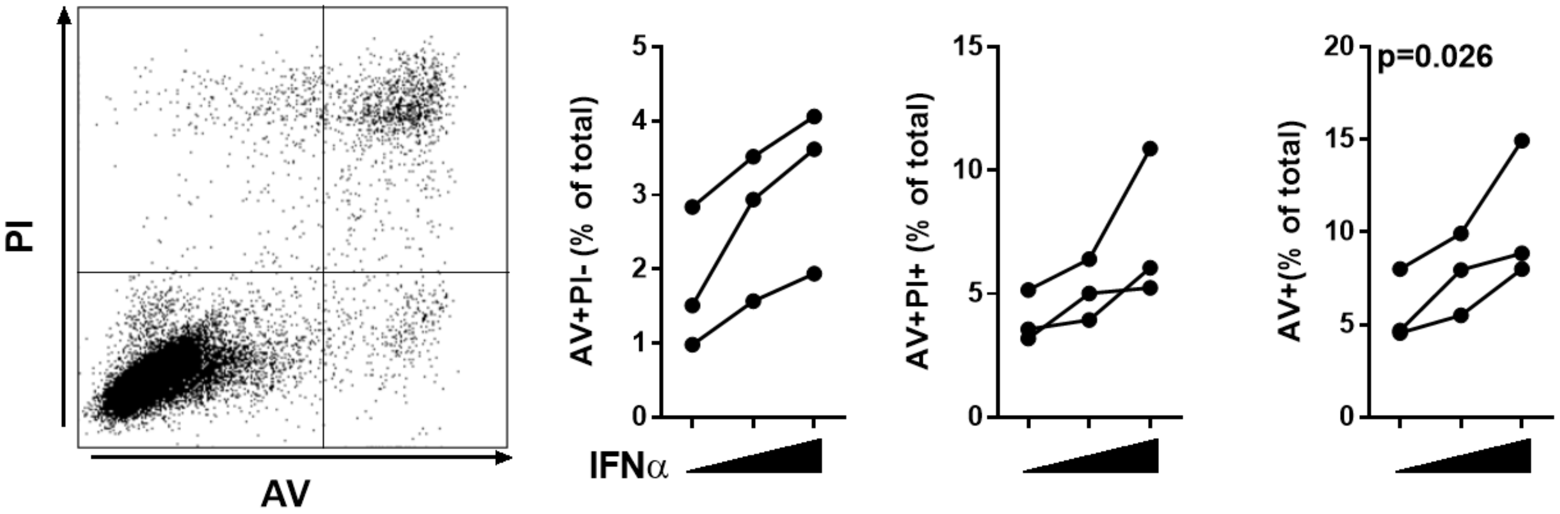


**Supplementary Figure 18. Treatment of erythroblasts cultured from cord blood with recombinant interferon alpha**. Erythroblasts from the cord blood of 3 controls were cultured in differentiation media for 72 hours, and then treated with different doses (0, 100 and 1000 IU/L) of recombinant interferon alpha (IFNα). Cells were gated according to the expression of propidium iodide (PI) and Annexin V (AV) as markers of apoptosis, and the effect of IFNα dose on apoptosis calculated ($p < 0.05$).

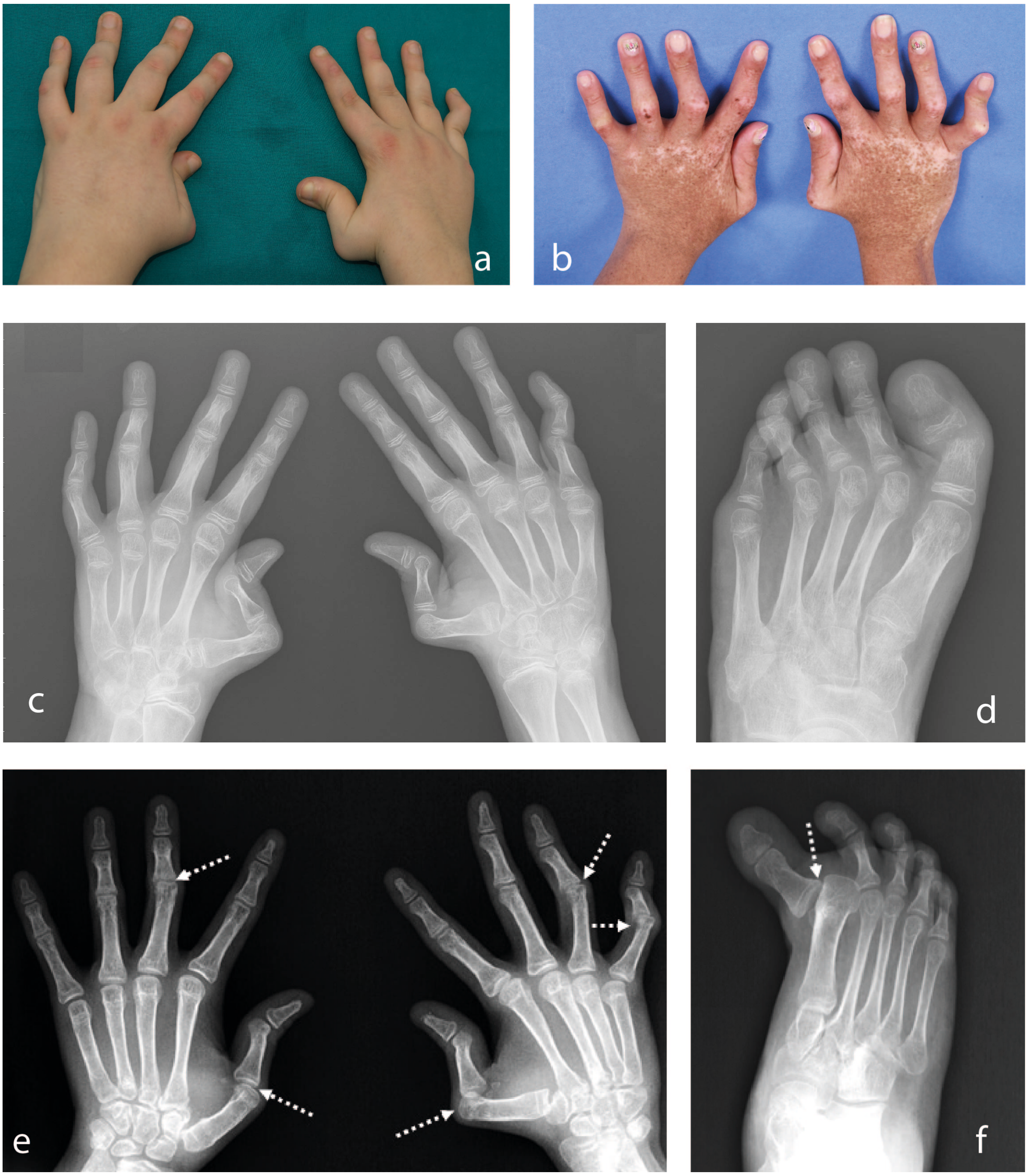


**Supplementary Figure 19. Overlap in the deforming arthropathy observed in F2:II-4 and patients with gain-of-function mutations in *IFIH1*.** Patient F2:II-4 developed a non-destructive, deforming arthropathy beginning at the age of 8 years (a, c, d). Similar features have been observed in patients with heterozygous gain-of-function mutations in *IFIH1*, encoding the cytosolic double-stranded RNA sensor MDA5 (b, e, f – 18 year old female with a c.992C>T (p.Thr331Ile) mutation in *IFIH1*)[2]. Note the subluxations (arrows in E and F) with well-preserved joint spaces and articular surfaces, reminiscent of Jaccoud's arthropathy. In all cases currently known to us, this phenotype is refractory to broad-spectrum immunosuppression, as well as anti-IL-1 receptor, anti-IL-1β and anti-TNFα therapy.

**Supplementary Table 1. Inflammatory and immunological indices in F1:V-1[a,b]**

| **Age (years)** | 0.8 | 2.3 | 7.4 | 7.6 | 8 | 8.4 | 8.6 | 9.3 | 10 | 10.3 | 10.5 |
|---|---|---|---|---|---|---|---|---|---|---|---|
| **Inflammatory markers** | | | | | | | | | | | |
| CRP (mg/L) | 6 | | 6 | 6 | 12 | 6 | 1.1 | 0.8 | < 0.5 | 0.8 | 0.8 |
| ESR (mm/h) | 26 | | 40 | 62 | 72 | 31 | 40 | 30 | 27 | 53 | 53 |
| IFN signature | | | | | | 1.747 | | 52.646 | | | 40.457 |
| **Autoantibodies** | | | | | | | | | | | |
| ANA | | 1:100 | | 1:100 | | | 1:400 | | | 1:160 | |
| Anti-DNA‡ (IU/mL) | | Neg | Neg | Neg | 43.4 | | 27.4 | | | 77 | > 100 |
| ANCA | | | | | | | | | | Neg | |
| Others | | Note 1 | | | | | | | | Note 2 | |
| **Immunoglobulins** | | | | | | | | | | | |
| IgG (g/L) | | | 17.82 | | | 17.17 | | 18.62 | 17.55 | 18.65 | |
| IgA (g/L) | | | 1.84 | | | 2 | | 1.82 | 1.76 | 1.98 | |
| IgM (g/L) | | | 0.7 | | | 0.8 | | 0.79 | 0.75 | 0.92 | |
| IgE (IU/L) | | | 247† | | | | | 67.3¶ | | | |
| **Immunophenotyping** | | | | | | | | | | | |
| Lymphocytes (/µL) | | | | 2100 | | 2800 | | | | 1945 | |
| T CD3$^+$ (/µL) | | | | 1512 | | 2324 | | | | 1459 | |
| T CD4$^+$ (/µL) | | | | 756 | | 896 | | | | 700 | |
| T CD8$^+$ (/µL) | | | | 693 | | 1400 | | | | 681 | |
| B cells (CD19$^+$) (/µL) | | | | 336 | | 392 | | | | 350 | |
| CD21$^{++}$CD24$^+$/CD19$^+$ (%) | | | | | | | | | | 71 | |
| CD27$^+$IgD$^+$/CD19$^+$ (%) | | | | | | | | | | 3 | |
| CD27$^-$IgD$^+$/CD19$^+$ (%) | | | | | | | | | | 88 | |
| CD24$^{++}$CD38$^{++}$/CD19$^+$ (%) | | | | | | | | | | 10 | |
| CD24$^{++}$CD38$^{++}$CD27$^-$IgD$^+$/CD19$^+$ | | | | | | | | | | 10 | |

| | | | | | | | | | | | |
|---|---|---|---|---|---|---|---|---|---|---|---|
| (%) | | | | | | | | | | | |
| $CD24^{-}CD38^{++}/CD19^{+}$ (%) | | | | | | | | | | 3 | |
| $CD21^{low}CD38^{low}/CD19^{+}$ (%) | | | | | | | | | | 1.5 | |
| $CD27^{+}CD19^{+}$ (%) | | | | | | | | | | 10 | |
| $IgD^{-}/CD27^{+}/CD19^{+}$ (%) | | | | | | | | | | 7 | |
| NK cells ($CD16^{+}56^{+}$)(/ µL) | | | | 210 | | 84 | | | | 78 | |

Abbreviations: ANA = Antinuclear antibody; ANCA = Anti-neutrophil cytoplasmic antibody; CRP = C-reactive protein (normal < 6 mg/L); ESR = Erythrocyte sedimentation rate (normal < 20 mm/h); IFN signature = Interferon signature (normal < 2.466); Ig = Immunoglobulin; Neg = Negative; NK = Natural killer

[a] Reference ranges for immunoglobulin levels according to age given in Supplementary Table 4
[b] Reference ranges for immunophenotyping data according to age given in Supplementary Table 5

‡Anti-DNA antibodies (Farr test) positive if > 20% or > 5.5 IU/mL
†Normal < 124 IU/L
¶Normal < 148 IU/L
Note 1: Anti-SSA, anti-SSB, anti-RNP, anti-histone, anti-thyroglobulin, anti-thyroperoxydase negative
Note 2: Anti-SSA, anti-SSB, anti-Sm, anti-RNP, anti-Scl70, anti-JO1 negative

**Supplementary Table 2. Inflammatory and immunological indices in F1:V-3[a,b]**

| **Age (years)** | 0.1 | 0.1 | 0.16 | 4 | 4.9 | 5.4 | 5.6 | 6 | 6.4 | 6.7 | 7 | 7.5 | 7.7** | 8 | 8.1 | 8.5 |
|---|---|---|---|---|---|---|---|---|---|---|---|---|---|---|---|---|
| **Inflammatory markers** | | | | | | | | | | | | | | | | |
| CRP (mg/L) | 6 | 6 | 6 | 28 | | | 6 | 6 | 6 | 6.9 | | 0.6 | 65.5 | <0.5 | 0.8 | < 0.5 |
| ESR (mm/h) | | | 62 | | | 85 | 76 | 116 | 40 | 51 | | 18 | 42 | | 40 | 23 |
| IFN signature | | | | | | | | 3.2 | 37.5 | | | 83.7 | 4.58 | | 14.4 | 4.23 |
| **Autoantibodies** | | | | | | | | | | | | | | | | |
| ANA | | | Neg | | | 1:10 | | Neg | | 1:10 | | | | | | |
| Anti-DNA‡ (IU/mL) | Pos | Pos | Neg | | | 43.8 | | | Neg | Neg | 9.6 | Neg | | | | |
| ANCA | | | | | | | | | | | | | | | | |
| Others | | | Note 1 | | | Note 2 | | Note 3 | | | | | | | | |
| **Immunoglobulins** | | | | | | | | | | | | | | | | |
| IgG (g/L) | | 8.21 | | 8.42 | 9.5 | 9.48 | 8.32 | 4.76 | | | 3.77 | 4.02 | 2.2 | 7.08 | 6.58 | 5.3 |
| IgA (g/L) | | 0.44 | | 1.58 | 1.6 | 1.78 | 1.69 | 1.52 | | | 0.6 | 0.44 | 0.17 | 0.75 | 0.35 | 0.48 |
| IgM (g/L) | | 0.79 | | 0.78 | 0.7 | 0.58 | 0.6 | 0.96 | | | 0.22 | 0.16 | 0.07 | 0.25 | 0.12 | 0.14 |
| IgE (IU/L) | | | | | | | 131 † | | | | | | | | | |
| **Immunophenotyping** | | | | | | | | | | | | | | | | |
| Lymphocytes (/μL) | 700 | 1400 | 2400 | 1700 | | | 1100 | 400 | | | 400 | 200 | | | | |
| T $CD3^+$ (/μL) | 539 | 1120 | 1728 | 1360 | | | 979 | | | | 292 | 152 | | | | |

| | | | | | | | | | | | | | | | | |
|---|---|---|---|---|---|---|---|---|---|---|---|---|---|---|---|---|
| T CD4$^+$ (/μL) | 280 | 546 | 792 | 595 | | | 440 | | | | 112 | 76 | | | | |
| T CD8$^+$ (/μL) | 245 | 504 | 840 | 731 | | | 517 | | | | 172 | 70 | | | | |
| CD4$^+$CD45RA$^+$CD31$^+$ (%) | | 47 | 56 | 52 | | | | | | | | | | | | |
| CD4$^+$CD45RO$^+$ (%) | | 15 | 20 | 25 | | | | | | | | | | | | |
| CD8$^+$CD45RA$^+$CCR7$^+$ (%) | | | | 95 | | | | | | | | | | | | |
| CD8$^+$CD45RA$^-$CCR7$^+$ (%) | | | | 1 | | | | | | | | | | | | |
| CD8$^+$CD45RA$^-$CCR7$^-$ (%) | | | | 2 | | | | | | | | | | | | |
| CD8$^+$CD45RA$^+$CCR7$^-$ | | | | 2 | | | | | | | | | | | | |
| B cells (CD19+) (/μL) | 70 | 84 | 600 | 272 | | | 66 | | | | 20 | 12 | | | | |
| NK cells (CD16$^+$56$^+$)(/μL) | 21 | 140 | 96 | 68 | | | 33 | | | | 88 | 36 | | | | |
| **T-cell proliferation (cpm/10$^3$)** | | | | | | | | | | | | | | | | |
| PHA | | 95.5 | | 45.5 | | | | | | | | | | | | |
| Candidine | | | | 13.4 | | | | | | | | | | | | |
| Tetanus toxoid | | | | 10 | | | | | | | | | | | | |

Abbreviations: ANA = Antinuclear antibody; ANCA = Anti-neutrophil cytoplasmic antibody; CRP = C-reactive protein (normal < 6 mg/L); ESR = Erythrocyte sedimentation rate (normal < 20 mm/h); IFN signature = Interferon signature (normal < 2.466); Ig = Immunoglobulin; Neg = Negative; NK = Natural killer; PHA = Phytohemagglutinin. Pos = Positive

[a] Reference ranges for immunoglobulin levels according to age given in Supplementary Table 4
[b] Reference ranges for immunophenotyping data according to age given in Supplementary Table 5

¶ Initiation of intravenous immunoglobulin therapy
‡ Anti-DNA antibodies (Farr test) positive if > 20% or > 5.5 IU/mL
† Normal < 98 IU/L
Note 1: Anti-SSA, anti-SSB, anti-RNP, anti-histone, anti-thyroglobulin, anti-thyroperoxydase negative
Note 2: Anti-Endomysium, anti-transglutaminase, anti-GAD65, anti-IA2, anti-islet, anti-ZNT8 negative
Note 3: Anti-IA2, anti-islet, anti-ZNT8 negative

**Supplementary Table 3. Inflammatory and immunological indices in F2:II-4[a,b]**

| **Age (years)** | 13.8 | 14.3 | 15.6 | 16.3 | 16.7 | 17.3 | 17.4 | 17.5 |
|---|---|---|---|---|---|---|---|---|
| **Inflammatory markers** | | | | | | | | |
| CRP (mg/L) | 71.1 | 10.7 | 21.6 | 11.6 | 9.5 | 10.0 | 1.5 | 4.5 |
| ESR (mm/h) | 58 | 22 | 78 | 30 | 21 | 9.0 | 5 | 12 |
| IFN signature | | | | 55.8 | 53.7 | 83 | 66 | 66 |
| **Autoantibodies** | | | | | | | | |
| ANA | Neg | | | | | Pos | | Neg |
| Anti-DNA[‡] (IU/mL) | 138 | 330 | | | | 30 | | |
| ANCA | Neg | | | | | | | |
| **Immunoglobulins** | | | | | | | | |
| IgG (g/L) | 10.9 | 8.1 | 9.3 | 9.2 | 9.0 | 8.5 | 8.6 | |
| IgA (g/L) | 1.3 | 0.8 | 0.9 | 1.2 | 1.3 | 1.2 | 1.4 | 1.5 |
| IgM (g/L) | 2.1 | 0.5 | 1.9 | 1.0 | 0.4 | 0.4 | 0.4 | |
| **Immunophenotyping** | | | | | | | | |
| Lymphocytes (#/mcL) | 300 | 400 | 600 | 600 | 300 | 640 | 900 | |
| Lymphocytes (%) | 19.2 | 7.7 | 13.4 | 14.2 | | | | 50.6 |
| T $CD3^+$ (%) | 81.5 | 73.6 | 84.9 | 84.6 | | | | 81.2 |
| T $CD4^+$ (%) | 51.6 | 56.2 | 33.3 | 62.5 | | | | 45.8 |
| T $CD8^+$ (%) | 43.1 | 38.5 | 55.8 | 31.1 | | | | 43.1 |
| B cells ($CD19^+$) (%) | 6.9 | 10.1 | 3.1 | 2.2 | | | | 0.8 |
| $CD27^+CD19^+$ (%) | 7.6 | 2.6 | 16.6 | | | | | |
| $CD27^+/IgM^+IgD^+/CD19^+$ (%) | 3.5 | 1.6 | 3.4 | | | | | |
| $CD27^-/IgM^+IgD^+/CD19^+$ (%) | 89.3 | 93.2 | 77.1 | | | | | |
| $CD27^+/ IgM^-IgD^-/CD19^+$ (%) | 4.1 | 1.0 | 13.2 | | | | | |
| $CD24^{++}CD38^{++}/CD19^+$ (%) | | 1.3 | 7.9 | | | | | |
| NK cells ($CD16^+56^+$)(%) | 11.5 | 14.2 | 9.6 | 10.7 | | | | 17.3 |

Abbreviations: ANA = Antinuclear antibody; ANCA = Anti-neutrophil cytoplasmic antibody; CRP = C-reactive protein (normal < 6 mg/L); ESR = Erythrocyte sedimentation rate (normal < 20 mm/h); IFN signature = Interferon signature (normal < 2.466); Ig = Immunoglobulin; Neg = Negative; NK = Natural killer

[a] Reference ranges for immunoglobulin levels according to age given in Supplementary Table 4
[b] Reference ranges for immunophenotyping data according to age given in Supplementary Table 5

‡Anti-DNA antibodies (Farr test) positive if > 20% or > 5.5 IU/mL

**Supplementary Table 4. Reference ranges for immunoglobulin levels according to age[3]**

| | Birth | 1 m | 3 m | 6 m | 1 y | 3 y | 5 - 9 y | 15 y | Adult |
|---|---|---|---|---|---|---|---|---|---|
| **IgG** | 6.1 - 13 | 4.6 - 8.6 | 2.9 - 5.5 | 2.3 - 4.4 | 3.3 - 6.2 | 4.8 - 8.9 | 5.5 - 11.5 | 6.5 - 12.3 | 6.6 - 12.8 |
| **IgA** | 0 - 0.2 | 0.1 - 0.3 | 0.1 - 0.4 | 0.2 - 0.6 | 0.2 - 0.8 | 0.3 - 1.2 | 0.4 - 1.6 | 0.5 - 2 | 0.7 - 3.4 |
| **IgM** | 0.04 - 0.6 | 0.2 - 0.7 | 0.3 - 0.8 | 0.3 - 0.9 | 0.5 - 1.3 | 0.5 - 1.5 | 0.5 - 1.5 | 0.5 - 1.6 | 0.5 - 2.1 |

Abbreviations: Ig = Immunoglobulin; m = Month; y = Year

**Supplementary Table 5. Reference ranges for immunophenotyping data according to age[4]**

| | 0-3 m | 3-6 m | 6-12 m | 1-2 y | 2-6 y | 6-12 y | 12- 18 y |
|---|---|---|---|---|---|---|---|
| Lymphocytes ($10^{-3}$/µL) | 5. 40 (3.40 - 7.60) | 6.30 (3.90 - 9.00) | 5.90 (3.40 - 9.00) | 5.50 (3.60 - 8.90) | 3.60 (2.30 - 5.40) | 2.70 (1.90 - 3.70) | 2.20 (1.40 - 3.30) |
| T CD3$^+$($10^{-3}$/µL) | 3.68 (2.50 - 5.50) | 3.93 (2.50 - 5.60) | 3.93 (1.90 - 5.90) | 3.55 (2.10 - 6.20) | 2.39 (1.40 - 3.70) | 1.82 (1.20 - 2.60) | 1.48 (1.00 - 2.20) |
| T CD4$^+$($10^{-3}$/µL) | 2.61 (1.60 - | 2.85 (1.80 - 4.00) | 2.67 (1.40 - 4.30) | 2.16 (1.30 | 1.38 (0.70 | 0.98 (0.65 | 0.84 (0.53 |

| | | | | | | | |
|---|---|---|---|---|---|---|---|
| | 4.00) | | | - 3.40) | - 2.20) | - 1.50) | - 1.30) |
| T $CD8^{+}$($10^{-3}$/μL) | 0.98 (0.56 - 1.70) | 1.05 (0.59 - 1.60) | 1.04 (0.50 - 1.70) | 1.04 (0.62 - 2.00) | 0.84 (0.49 - 1.30) | 0.68 (0.37 - 1.10) | 0.53 (0.33 - 0.92) |
| $CD4^{+}CD45RA^{+}CD31^{+}$(%) | | | | | | | |
| $CD4^{+}CD45RO^{+}$ (%) | 10 (2 - 22) | 8 (3 - 16) | 9 (5 - 18) | 12 (7 - 20) | 16 (9 - 26) | 21 (13 - 30) | 28 (18 - 38) |
| $CD8^{+}CD45RA^{+}CCR7^{+}$(%) | | | | | | | |
| $CD8^{+}CD45RA^{-}CCR7^{+}$(%) | | | | | | | |
| $CD8^{+}CD45RA^{-}CCR7^{-}$ (%) | | | | | | | |
| $CD8^{+}CD45RA^{+}CCR7^{-}$ | | | | | | | |
| B cells ($CD19^{+}$) ($10^{-3}$/mL) | 0.73 (0.30 - 2.00) | 1.55 (0.43 - 3.00) | 1.52 (0.61 - 2.60) | 1.31 (0.72 - 2.60) | 0.75 (0.39 - 1.40) | 0.48 (0.27 - 0.86) | 0.30 (0.11 - 0.57) |
| $CD21^{++}CD24^{+}/CD19^{+}$ (%) | | | | | | | |
| $CD27^{+}IgD^{+}/CD19^{+}$ (%) | | | | | | | |
| $CD27^{-}IgD^{+}/CD19^{+}$ (%) | | | | | | | |
| $CD24^{++}CD38^{++}/CD19^{+}$ (%) | | | | | | | |
| $CD24^{++}CD38^{++}CD27^{-}IgD^{+}/CD19^{+}$ (%) | | | | | | | |
| $CD24^{-}CD38^{++}/CD19^{+}$ (%) | | | | | | | |
| $CD21^{low}CD38^{low}/CD19^{+}$ (%) | | | | | | | |
| $CD27^{+}CD19^{+}$ (%) | | | | | | | |
| $IgM^{-}IgD^{-}/CD27^{+}/CD19^{+}$ (%) | | | | | | | |
| $IgD^{-}/CD27^{+}/CD19^{+}$ (%) | | | | | | | |
| NK cells ($CD16^{+}56^{+}$) ($10^{-3}$/μL) | 0.42 (0.17 - 1.10) | 0.42 (0.17 - 0.83) | 0.40 (0.16 - 0.95) | 0.36 (0.18 - 0.92) | 0.30 (0.13 - 0.72) | 0.23 (0.10 - 0.48) | 0.19 (0.07 - 0.48) |

Values are presented as medians (10th and 90th percentiles). Abbreviations: Ig = Immunoglobulin; m = Month; NK = Natural killer; y = Year

**Supplementary Table 6. *In silico* predictions of pathogenicity associated with the identified *DNASE2* homozygous variants**

| | | |
|---|---|---|
| **Genomic** | Chr19(GRCh37):g.12989648C>G | Chr19(GRCh37):g.12989633T>A |
| **cDNA** | c.347G>C | c.362A>T |
| **Protein** | p.Gly116Ala | p.Asp121Val |
| **ExAc frequency** | Novel (107184) | Novel (109840) |
| **gnomAD frequency** | Novel (246270) | Novel (275830) |
| **SIFT** | Deleterious (0.01) | Deleterious (0) |
| **Polyphen2** | Probably damaging (1.00) | Probably damaging 0.989 |
| **Mutation taster** | Disease causing (1) | Disease causing (1) |
| **LoFtool** | Possibly damaging (0.497) | Possibly damaging (0.497) |
| **CADD phred** | Deleterious (26.1) | Deleterious (24.3) |
| **Condel** | Deleterious (0.905) | Deleterious (0.892) |
| **MaxEnt** | -19.1%† | 0.0% |
| **NNSPLICE** | -17.6%† | -3.1% |
| **HSF** | -4.5%† | 0.0% |

† The G at base 347 of the cDNA is the middle nucleotide of the 116th codon and the first nucleotide of exon 4, and therefore predicted to change an acceptor splice site.

ExAc browser Beta version (http://exac.broadinstitute.org) and gnomAD browser beta version (http://gnomad.broadinstitute.org) accessed on 06/01/2017.

**Supplementary Table 7. Details of samples investigated for whole genome RNA expression**

| | **Gene (mutations)** | **Interferon score (age*)** |
|---|---|---|
| **F1:V-1** | *DNASE2* (Hom: c.374G>C / p.Gly116Ala | 52.64 (9.28) |
| **F1:V-3** | *DNASE2* (Het: c.374G>C / p.Gly116Ala | 37.58 (6.4) & 14.44 (8.27) |
| **F831_3** | *TMEM173* (Het: c.463G>A / p.V155M) | 8.69 (65.31) |
| **F1802** | *TMEM173* (Het: c.617G>A / p.Cys206Tyr) | 6.56 (16.12) |
| **F1975** | *TMEM173* (Het: c.463G>A / p.V155M) | 14.07 (12.48) |
| **F441** | *TREX1* (Hom: c.868_885 del/ p.Pro290_Ala295 del) | 19.791 (4.64) |
| **F350** | *TREX1* (Hom: c.393_408dup / p.Glu137Profs*24 ) | 18.187 (6.34) |

* Decimalized age in years

**Supplementary Table 8. Bone marrow findings in F1:V-3**

| **Age (years)** | Birth | 4.9 | 6 | 7.8 | 8.5 |
|---|---|---|---|---|---|
| **Blood** | NA | Mild poikilocytosis | Poikilocytosis, anisocytosis, anisochromia | NA | Mild poikilocytosis with normal platelet morphology |
| **Bone marrow** | Hemodilution | Rich cellularity; normal abundance and morphology of megakaryocytes, myeloid and erythroid cells with no excess of blasts | Mild cellularity; mild reduction of megakaryocytes; decreased myeloid precursors; features of dyserythropoeisis in late-stage erythroblasts with no excess of blasts | Decreased cellularity; few megakaryocytes (with those present having normal morphology); hypoplasia of the myeloid compartment; features of dyserythropoeisis in late-stage erythroblasts with no excess of blasts | NA |
| **Perl's staining** | No ringed sideroblasts | No ringed sideroblasts | No ringed sideroblasts | No ringed sideroblasts | NA |

Abbreviations: NA = Not assessed

## Supplementary references


1. Itan, Y. *et al.* The mutation significance cutoff: gene-level thresholds for variant predictions. *Nat Methods* **13**, 109-10 (2016).
2. de Carvalho, L.M. *et al.* Musculoskeletal Disease in MDA5-Related Type I Interferonopathy: A Mendelian Mimic of Jaccoud's Arthropathy. *Arthritis Rheumatol* **doi: 10.1002/art.40179.**(2017).
3. Picard, C. [How to diagnose a hereditary immunodeficiency?]. *Rev Prat* **57**, 1671-6 (2007).
4. Shearer, W.T. *et al.* Lymphocyte subsets in healthy children from birth through 18 years of age: the Pediatric AIDS Clinical Trials Group P1009 study. *J Allergy Clin Immunol* **112**, 973-80 (2003).
